# Genome-Wide Identification of Targets and Function of Individual MicroRNAs in Mouse Embryonic Stem Cells

**DOI:** 10.1371/journal.pgen.1001163

**Published:** 2010-10-21

**Authors:** Sophie A. Hanina, William Mifsud, Thomas A. Down, Katsuhiko Hayashi, Dónal O'Carroll, Kaiqin Lao, Eric A. Miska, M. Azim Surani

**Affiliations:** 1 Wellcome Trust/Cancer Research UK Gurdon Institute, University of Cambridge, Cambridge, United Kingdom; 2 The Laboratory for Lymphocyte Signaling and the Laboratory of Molecular Immunology, The Rockefeller University, New York, New York, United States of America; 3 Molecular Cell Biology Division, Applied Biosystems, Foster City, California, United States of America; University of California San Francisco, United States of America

## Abstract

Mouse Embryonic Stem (ES) cells express a unique set of microRNAs (miRNAs), the miR-290-295 cluster. To elucidate the role of these miRNAs and how they integrate into the ES cell regulatory network requires identification of their direct regulatory targets. The difficulty, however, arises from the limited complementarity of metazoan miRNAs to their targets, with the interaction requiring as few as six nucleotides of the miRNA seed sequence. To identify miR-294 targets, we used *Dicer1-*null ES cells, which lack all endogenous mature miRNAs, and introduced just miR-294 into these ES cells. We then employed two approaches to discover miR-294 targets in mouse ES cells: transcriptome profiling using microarrays and a biochemical approach to isolate mRNA targets associated with the Argonaute2 (Ago2) protein of the RISC (RNA Induced Silencing Complex) effector, followed by RNA–sequencing. In the absence of *Dicer1*, the RISC complexes are largely devoid of mature miRNAs and should therefore contain only transfected miR-294 and its base-paired targets. Our data suggest that miR-294 may promote pluripotency by regulating a subset of *c-Myc* target genes and upregulating pluripotency-associated genes such as *Lin28*.

## Author Summary

Stem cells in plants and animals contain many small RNAs, which help to regulate differentiation into diverse cell types. Mutation in a gene necessary for the maturation of small RNAs in plants causes the stem cells (called meristem cells) to remain in an indeterminate, overproliferating state. Similarly in worms, a small RNA called *lin-4* miRNA prevents “stem cell–like cells” appearing at inappropriate times. Thus, it is important to determine the precise functions of key individual small RNAs in embryonic stem cells. To address this, we created embryonic stem cells lacking all miRNAs into which we introduced a single miRNA. We discovered that a single miRNA could affect the expression of many genes in stem cells, which in turn regulate key properties of stem cells. These together help establish an intricate network of gene regulation in stem cells that defines their properties. Our findings are of broad interest because different miRNAs have critical functions in diverse cell types in developing embryos. It is important to understand the function of these molecules also because misregulation of miRNA function underlies some human diseases, including cancers.

## Introduction

Embryonic stem cells, which are derived from the inner cell mass of the blastocyst, hold great clinical promise because of their unique capacity to both self-renew and differentiate into potentially any cell type. Understanding the molecular controls of pluripotency is key to realising their therapeutic potential. While the general importance of small RNAs in gene regulation has been recognised in plants and animals, much remains to be understood about the specific role of small RNAs in ES cells.

miRNAs and small interfering RNAs (siRNAs) are a class of small (≈20–25 nucleotide) non-coding RNAs that direct sequence-specific post-transcriptional repression of target mRNAs. Mature miRNAs and siRNAs are generated from double stranded RNA (dsRNA) precursors by the RNase III enzyme Dicer [1], [2]. The mature small RNA is then incorporated into a protein of the Argonaute family [3], [4]. This RNA-protein complex forms the core of the effector complex referred to as the RNA-induced silencing complex (RISC). Within the RISC, the small RNA acts a guide to direct Argonaute proteins to complementary target transcripts to elicit the cleavage, degradation or translational repression of their targets depending on their degree of complementarity [5].

**Figure 1:**
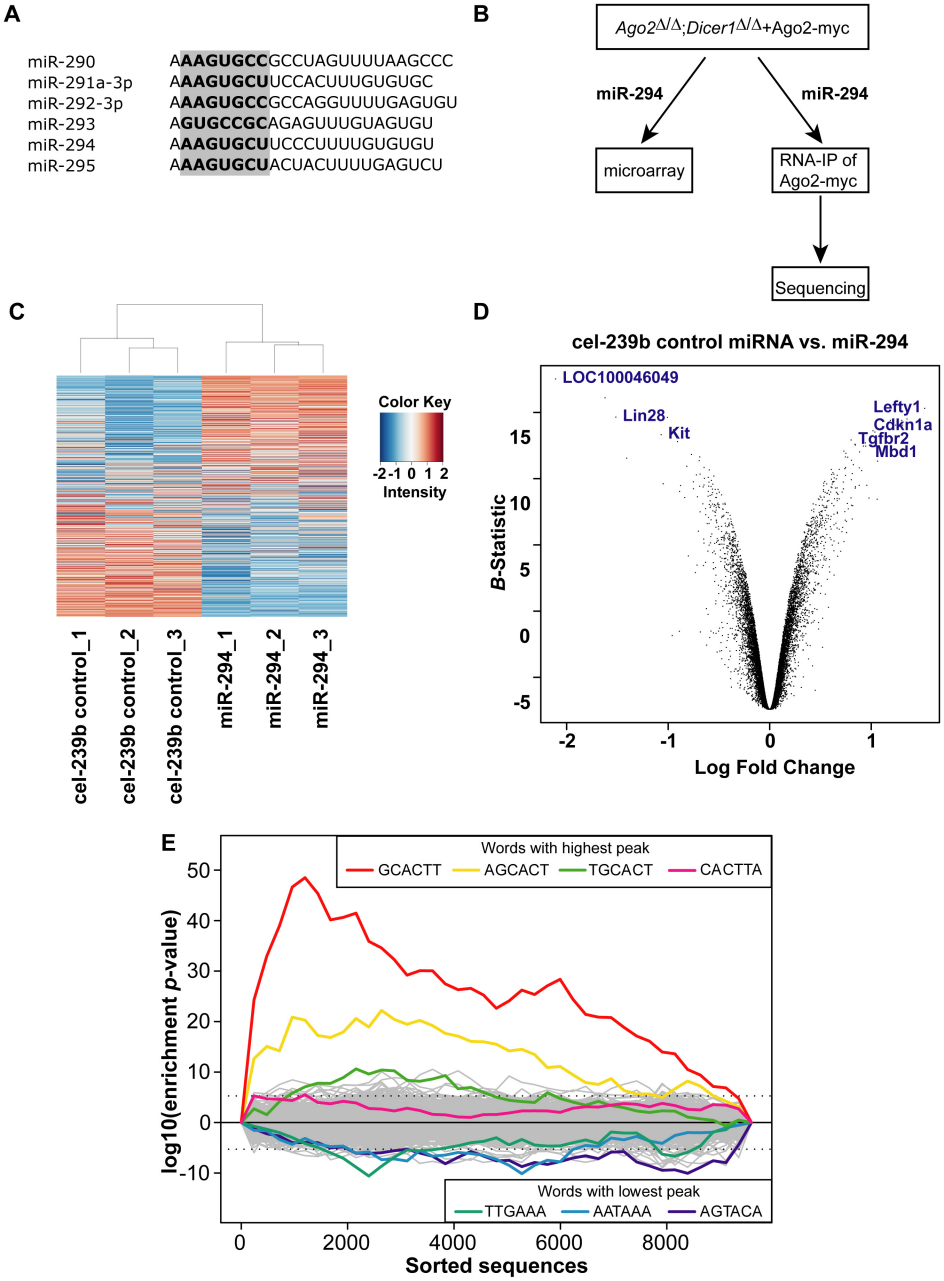
Microarray analysis of *Dicer1*-null ES cells transfected with miR-294. (A) Sequences of predominantly expressed mature miRNAs from the miR-290-295 cluster with their seed sequence highlighted in bold. (B) Experimental scheme to identify miR-294 targets: miR-294 was transfected into *Ago2* ^Δ/Δ^;*Dicer1* ^Δ/Δ^ ES cells transgenic for Ago2-myc; the transcriptome profile was analysed by microarray; and miR-294-programmed Ago2-myc was subjected to RNA-immunoprecipitation and deep sequencing of the bound mRNA. (C) Heatmap showing differentially expressed genes between miR-294-transfected and cel-239b-transfected ES cells. The heat map was colour coded using red for downregulation and blue for upregulation (with respect to the mean signal in all the six samples). (D) Volcano plot to set threshold for differentially expressed genes. A negative log Fold Change on the *x*-axis corresponds to upregulated genes in miR-294 transfected cells whereas, a positive log Fold Change corresponds to downregulated genes in miR-294 transfected cells. (E) Overrepresentation of miR-294 seed matches in the most downregulated genes of the microarray. The motif-discovery tool Sylamer was used. The *x*-axis represents the sorted gene list from most downregulated (left) to most upregulated (right). The *y*-axis shows the significance for each word.

Several studies implicate miRNAs in the control of early embryonic development and maintenance of the pluripotent stem cell state. Disruption of the single *Dicer1* gene in mice leads to early embryonic lethality around E7.5 [6]. *Dicer1* mutant embryos have greatly reduced expression of *Oct-4* in the epiblast, implying a lack of pluripotent cells, and it is not possible to derive ES cells from *Dicer1*-null blastocysts. However, conditional deletion of *Dicer1* from established ES cells results in an impaired capacity to differentiate, as well as a profound initial proliferation defect that is overcome with time [7], [8]. Moreover, large scale cloning and sequencing efforts have revealed a subset of miRNAs that are unique to ES cells [9]–[11]. The miR-290-295 cluster (Figure 1A) consists of 6 miRNAs that share a similar 5′ region from nucleotides 2–8, known as the ‘seed’ sequence, which is thought to be the primary specificity determinant for target recognition in most miRNAs [12]. The miR-290-295 cluster accounts for the majority of all miRNAs expressed in undifferentiated ES cells but decreases after ES cells differentiate [9]. Recent evidence suggests that there are functional differences between miRNAs from the miR-290-295 cluster. Only miR-291-3p, miR-294 and miR-295 can promote the G1-S transition of the cell cycle and the induction of pluripotency [13], [14]. Furthermore, miR-293 expression and seed sequence differs markedly from the other members of this family, indicating the need to re-examine previous inferences based on whole miR-290-295 overexpression studies [15]–[17].

To identify targets and study the function of individual miRNAs in ES cells, we used *Dicer1*-null ES cells in which we replaced endogenous Ago2 with a transgenic Ago2-myc (*Dicer1* ^Δ/Δ^+Ago2-myc) (Figure 1B). In this *Dicer1*-null background, there are no mature miRNAs [11], which enables the reintroduction of any single miRNA at a time. To investigate the role of miR-294 in ES cells, we profiled the transcriptome of *Dicer1*-null ES cells transfected with miR-294. In addition, we used a biochemical approach that exploits the direct interaction of the mature miRNA to its mRNA target within the Ago protein of the RISC complex [18]. Immunoprecipitation of Ago2-myc from *Dicer1*-null ES transfected with miR-294, followed by RNA-sequencing of associated RNAs should lead to identification of direct miR-294 targets. Our strategy overcomes the problem of immunoprecipitating RISC complexes that contain many different miRNAs with their corresponding targets [19]–[21]. This approach is applicable to the study of any single miRNA or combination of miRNAs, potentially in any cell type. We describe here the results of the microarray and RNA-Sequencing analyses, and how miR-294 integrates into the ES cell regulatory network.

## Results

### Transcriptome analysis of miR-294–transfected *Dicer1*-null ES cells

First, we established *Dicer1*-null ES cells that lack all mature miRNAs (Figure S1B, S1C), into which we can reintroduce one specific miRNA of interest at a time. Previous studies have shown that the miR-290-295 cluster might have an important role in the pluripotency of ES cells [14], [16], [17] amongst which miR-294 is one of the most abundantly expressed miRNAs (Figure S1A) [11]. We decided to investigate the potential role of miR-294 in pluripotency.

Metazoan miRNAs typically bind to partially complementary sites in the 3′ untranslated region (3′ UTRs) of their target mRNAs to direct translational repression or mRNA destabilisation [22]–[24]. Overexpression of a miRNA can affect hundreds of mRNAs and messages that are downregulated tend to have significant enrichment of sequences complementary to the corresponding seed of the miRNA [16], [17], [24]. To identify the global effects of miR-294 in ES cells, we analysed the transcriptomes of *Dicer1* ^Δ/Δ^+Ago2-myc ES cells transfected with miR-294 compared to *Dicer1* ^Δ/Δ^+Ago2-myc ES cells transfected with a cel-239b control miRNA, which has minimal sequence identity to mouse miRNAs. The transcriptomes were profiled using Illumina microarrays (GEO accession code: GSE20048). The relative signal intensities of each of the 15735 probes across the six samples were plotted as a heat map (Figure 1C), which revealed global downregulation and upregulation of probes in each sample transfected with miR-294. To select differentially expressed genes, probes were ranked by the log odds ratio (*B*-statistic) of each probe showing differential expression, and plotted against the log fold change in a ‘volcano’ plot (Figure 1D). A *B*-statistic >5 (and corresponding to an adjusted *p*-value <0.0001) was selected as a cut-off for differential expression (Figure 1C). Using this conservative cut-off gives, with very high confidence, 162 upregulated (Table S1) and 248 downregulated differentially expressed genes upon miR-294 transfection.

To determine whether the downregulated transcripts contain miR-294 seed matches, we used the k-mer composition analysis tool Sylamer [25] to search for overrepresentation of sequence motifs in the 3′ UTRs. Each gene on the array (for which a 3′ UTR sequence was annotated) was ranked from most downregulated to most upregulated according to log fold change. If enrichment of the seed sequence in the 3′ UTRs correlates with the ranking of genes according to their fold change, then one would expect to observe a sharp peak in overrepresentation of the miRNA seed for the top-ranked genes. The resulting enrichment analysis plot shows that a strong signal is evident for 6-mer words corresponding to the seed region of miR-294 (Figure 1E), peaking approximately at gene 1000 in the ranked list. Conversely, this sequence is depleted in upregulated genes. The maximum of the first enrichment peak can be chosen as a threshold, above which, genes can be considered candidate targets if they contain the appropriate seed sequences matches. This yielded a more relaxed list of 487 predicted targets, which have at least one 6-mer present in their 3′ UTR (Table S2). This Sylamer list contains all of the conservative list of predicted targets (127 out of 248 have at least one 6-mer in their 3′ UTR) selected using a *B*-statistic >5 (Table S2). In summary, there was overrepresentation of the miR-294 seed sequence in the most downregulated genes, indicating that many of the observed gene expression changes are likely to be consequences of miR-294 expression.

### RNA–IP and RNA–sequencing of Ago2-myc–bound mRNAs

To discover direct targets of miR-294, we used a biochemical approach to isolate mRNAs targeted by miRNAs within the Ago2 protein of the RISC. To facilitate the immunoprecipitation of Ago2, we replaced endogenous Ago2 with a myc-epitope tagged Ago2. We also constructed a catalytically-inactive mutant Ago2-myc (Ago2-myc-MUT) by point-mutating residues Q633R and H634A in the PIWI domain to capture the subset of target mRNAs that would otherwise be sliced by Ago2 [26], [27]. Point mutations Q633R and H634A were previously shown to abolish mouse Ago2 cleavage-activity without affecting siRNA binding [27]. First, we confirmed the specificity of Ago2-myc immunoprecipitation (IP) (Figure S2A), and that immunoprecipitation of Ago2-myc from *Ago2* ^flox/flox^;*Dicer1* ^flox/flox^ ES cells does retain miRNAs (Figure S2B). We then assessed the enrichment of known targets using *Dicer1*-null ES cells transfected with miR-294. We found enrichment of *Cdkn1a*, a known miR-294 target [13], in immunoprecipitated RNA from *Dicer1* ^Δ/Δ^+Ago2-myc ES cells transfected with miR-294, compared to cel-239b (Figure S2C). Following RNA-immunoprecipitation of miR-294-programmed Ago2-myc, RNA from the INPUT (total RNA) and IP (Figure S3A) were subjected to library preparation and sequenced by SOLiD (GEO accession code: GSE20199).

**Figure 2:**
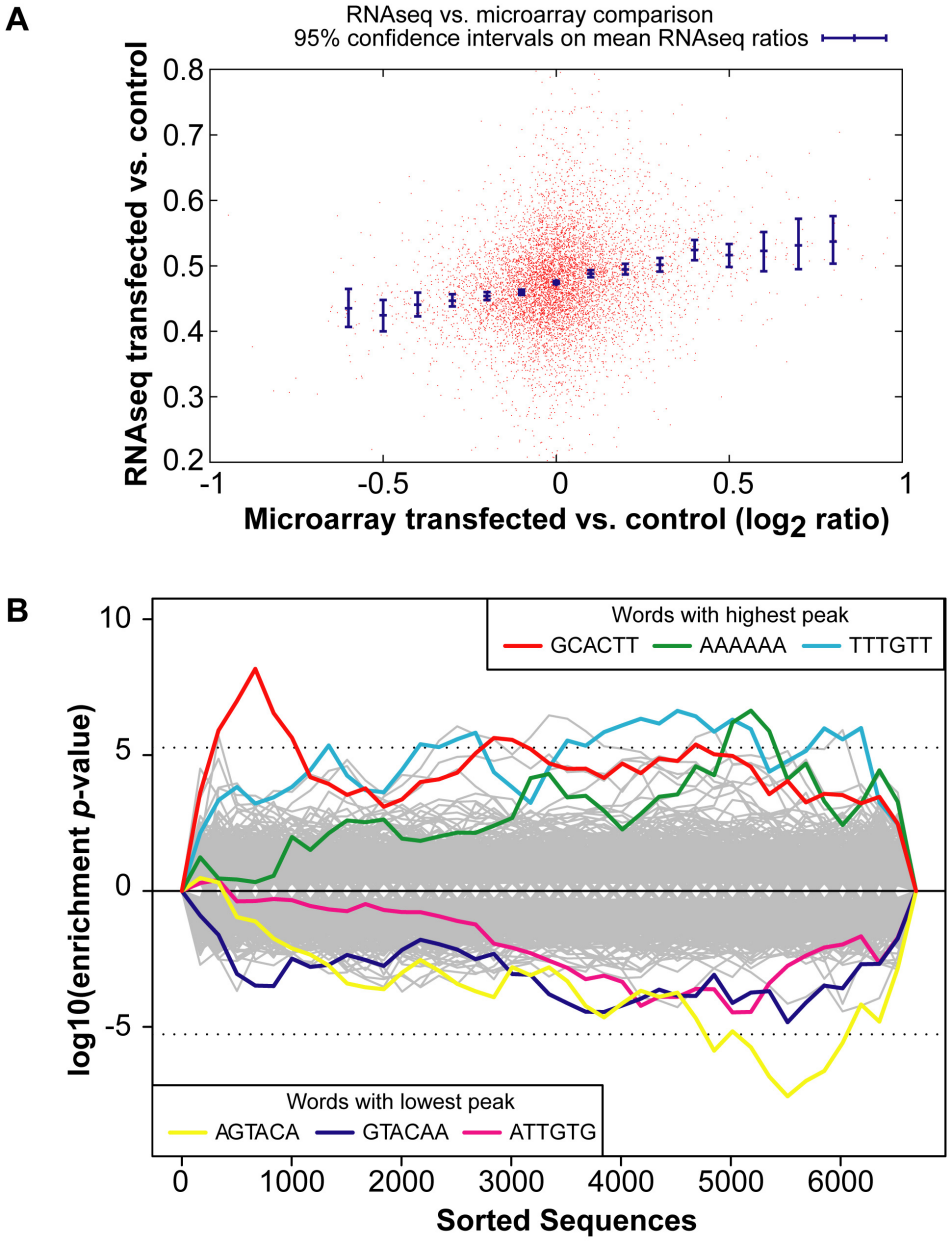
Global gene expression changes in the INPUT. (A) RNA-Sequencing (RNA-Seq) of the INPUT (Total RNA) follows the microarray trend in gene expression. The *y*-axis represents the RNA-Seq ratio of the INPUT from *Dicer1* ^Δ/Δ^+Ago2-myc ES cells transfected with miR-294 vs. cel-239b. Higher values mean enriched in cel-239b vs. miR-294. The *x*-axis represents the fold change in gene expression as measured by microarray. A positive value indicates upregulation of genes in miR-294-transfected cells and a negative value indicates downregulation of genes in miR-294-transfected cells. (B) Overrepresentation of 6-mer (position 2-7) seed matches to miR-294 in the INPUT of *Dicer1* ^Δ/Δ^+Ago2-myc ES cells transfected with miR-294. The *x*-axis represents the sorted gene list from most downregulated (left) to most upregulated (right). The *y*-axes show the significance for each word.

**Figure 3:**
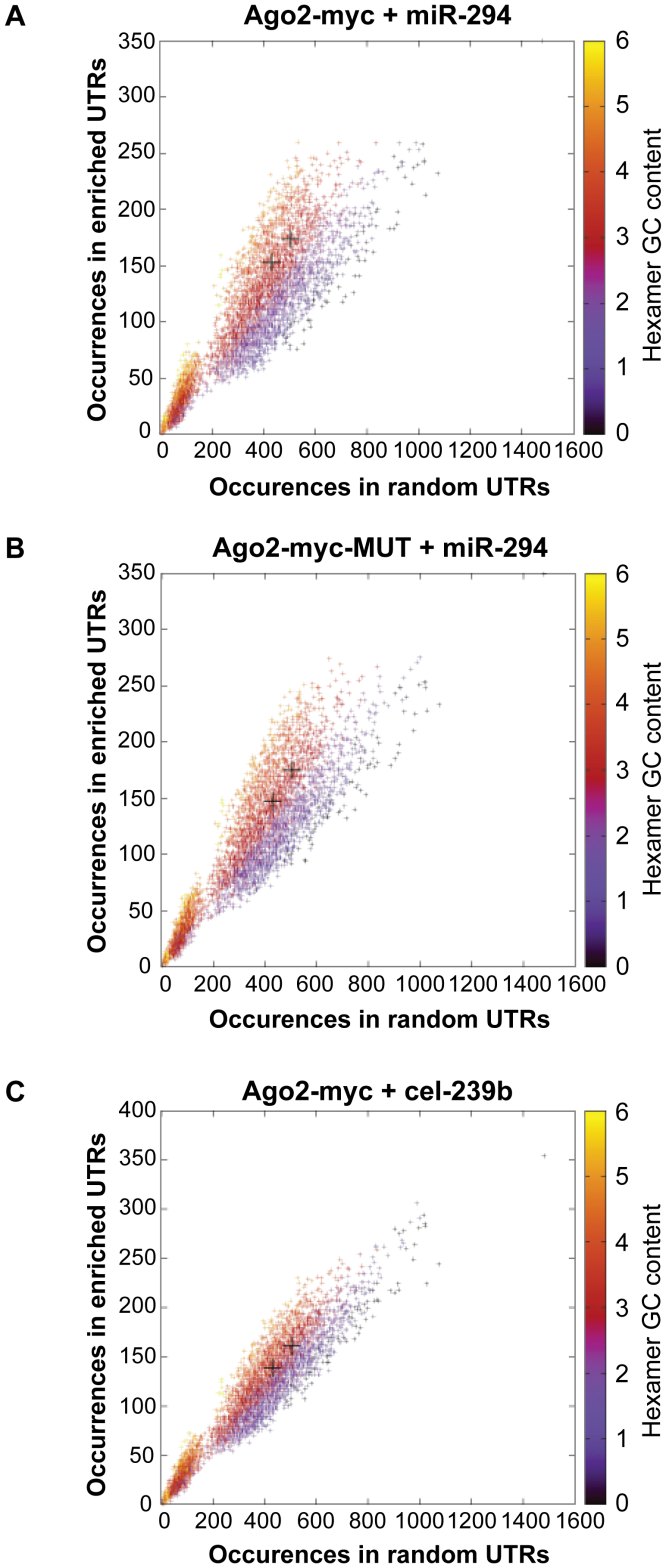
Hexamer composition of enriched 3′ UTRs. Each point represents a hexamer and the color corresponds to its GC content. This plot compares the frequency of hexamers present in ≈500 top-enriched 3′ UTRs vs. ≈2000 non-enriched 3′ UTRs for (A) Ago2-myc transfected with miR-294 (B) Ago2-myc-MUT transfected with miR-294 (C) Ago2-myc transfected with cel-239b. The black crosses indicate the three hexamers contained within the miR-294 seed 8-mer.

A comparison of the global gene expression changes detected by microarray and by RNA-Sequencing of the INPUT revealed a similar trend (Figure 2A). Furthermore, there was overrepresentation of the miR-294 seed sequence in the most downregulated genes of the sequenced INPUT (Figure 2B), indicating that miR-294 was functional and that the transfection efficiency was sufficiently high (Figure S3B) to bring about gene expression changes. An examination of the IP vs. INPUT ratios for each sample revealed an overall greater dynamic range of enrichment for samples transfected with miR-294 compared to cel-239b (Figure S4). The pattern was similar for the catalytically-inactive Ago2-myc. This result was expected since miRNA-directed slicing of the target mRNA is thought to occur rarely in metazoans [12]. However, the overall extra enrichment could not be accounted for by the overrepresentation of miR-294 seed sequence matches in the 3′ UTRs of enriched genes (Figure 3A). There is a surprising general tendency for hexamers with a higher GC content to be overrepresented in the 3′ UTRs of enriched transcripts (Figure 3A and 3B). This effect is also observed in samples transfected with the control cel-239b (Figure 3C). However, the GC effect is markedly stronger in the samples transfected with miR-294.

**Figure 4:**
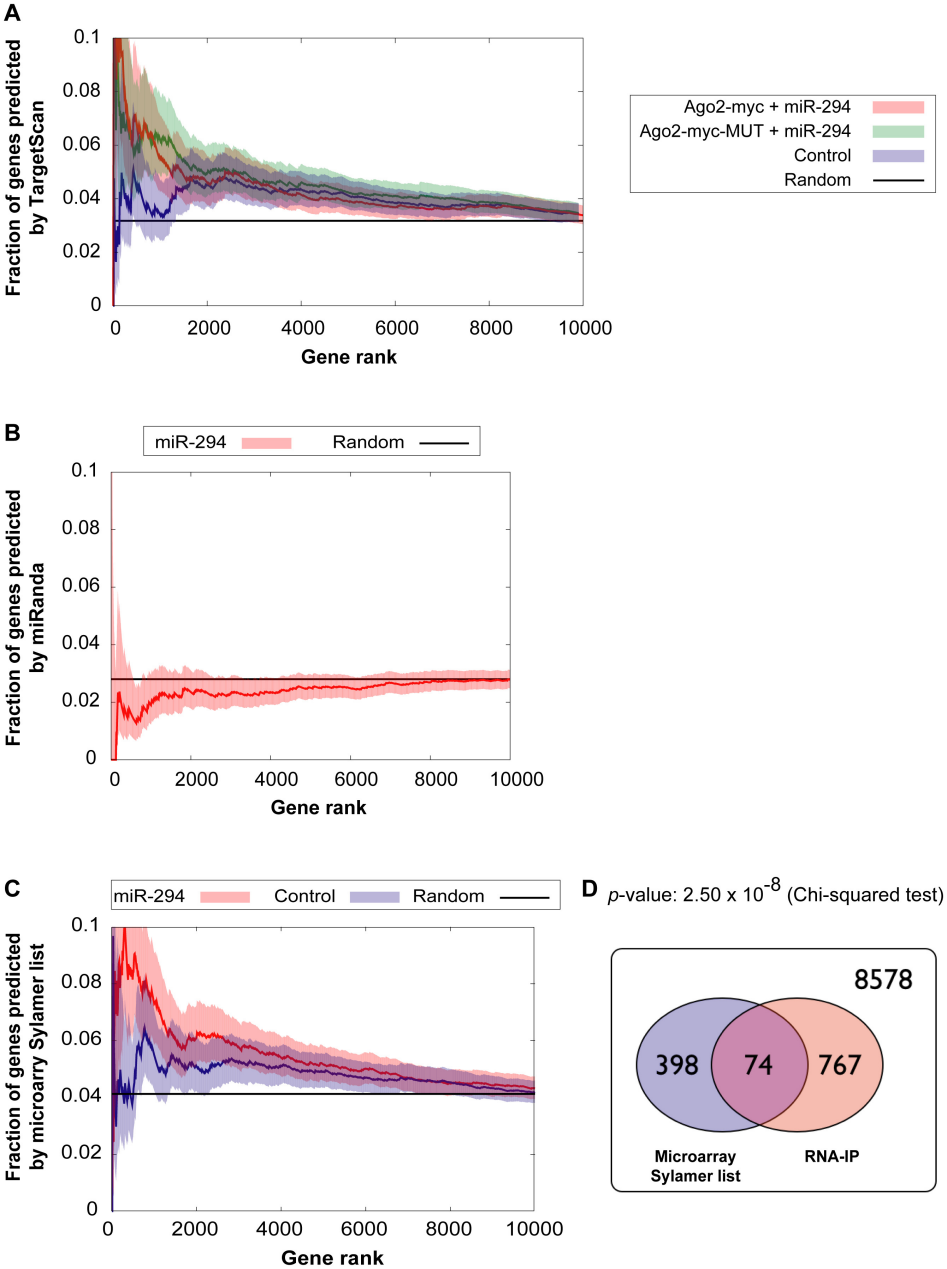
Comparison of genes enriched in the IP with computational and microarray predicted targets. (A,B) Correlation between enriched genes in the IP and computational target predictions. The *x*-axes represent fraction of genes ranked by increasing *p*-value for the likelihood of enrichment in the IP. The *y*-axes represent the fraction of those genes, which have a (A) TargetScan prediction (B) miRanda prediction. Red indicates Ago2-myc samples transfected with miR-294; green indicates Ago2-myc-MUT samples transfected with miR-294; blue indicates samples transfected with cel-239b control miRNA. The black line shows the expected outcome if enrichment and target predictions are independent from one another. (C) Correlation between enriched genes in the IP and microarray target predictions (selected from the Sylamer-based cut-off). The *x*-axis represents the fraction of genes ranked by *p*-value for the likelihood of enrichment in the IP. The threshold for selecting enrichment was a *p*-value <0.003 from the binomial model. (D) Venn diagram overlap between Sylamer microarray target predictions and enriched genes in the IP. 8578 represents the number of genes which were considered (both microarray and RNA-IP data was available) but which were neither depleted on the array nor enriched in the IP.

To ascertain whether this extra IP enrichment contains miR-294 targets, the correlation between our data and computational miR-294 target predictions was tested. The fraction of genes ranked by *p*-value (based on the likelihood of enrichment) that have a TargetScan prediction was calculated. For samples transfected with miR-294, approximately one-tenth (0.1) are predicted targets by TargetScan for the top 1000-most enriched genes (Figure 4A). For samples transfected with cel-239b, the cumulative fraction is considerably lower (0.04–0.06) (Figure 4A). Thus, there is a correspondence between the degree of observed enrichment and the TargetScan computational target predictions. In contrast, there was no correlation with miRanda [28] target predictions (Figure 4B).

To have a genome-wide benchmark for identifying miR-294 targets, we compared enriched genes in the IP (selected using a *p*-value <0.003 cut-off) with our microarray-predicted targets. There is a statistically significant correlation between IP enrichment and the microarray-predicted targets (Figure 4C and 4D). The trend is similar to the correlation between IP enrichment and TargetScan predictions (Figure 4A). Thus, miR-294 targets are enriched in the IP but there is also a non-seed-match-specific binding effect that correlates with hexamer GC content.

In summary, a substantial proportion of enriched genes with miR-294 seed matches are not, however, detected in the microarray target predictions. These genes could be direct targets whose transcript levels remain unchanged by miR-294 overexpression and are therefore, not detected by microarray analysis. Alternatively, the basis of this extra enrichment could represent novel targets with non-canonical seed matches, non-specific associations, or technical limitations.

### miR-294 may promote pluripotency through regulation of shared c-Myc targets and upregulation of Lin28

**Figure 5:**
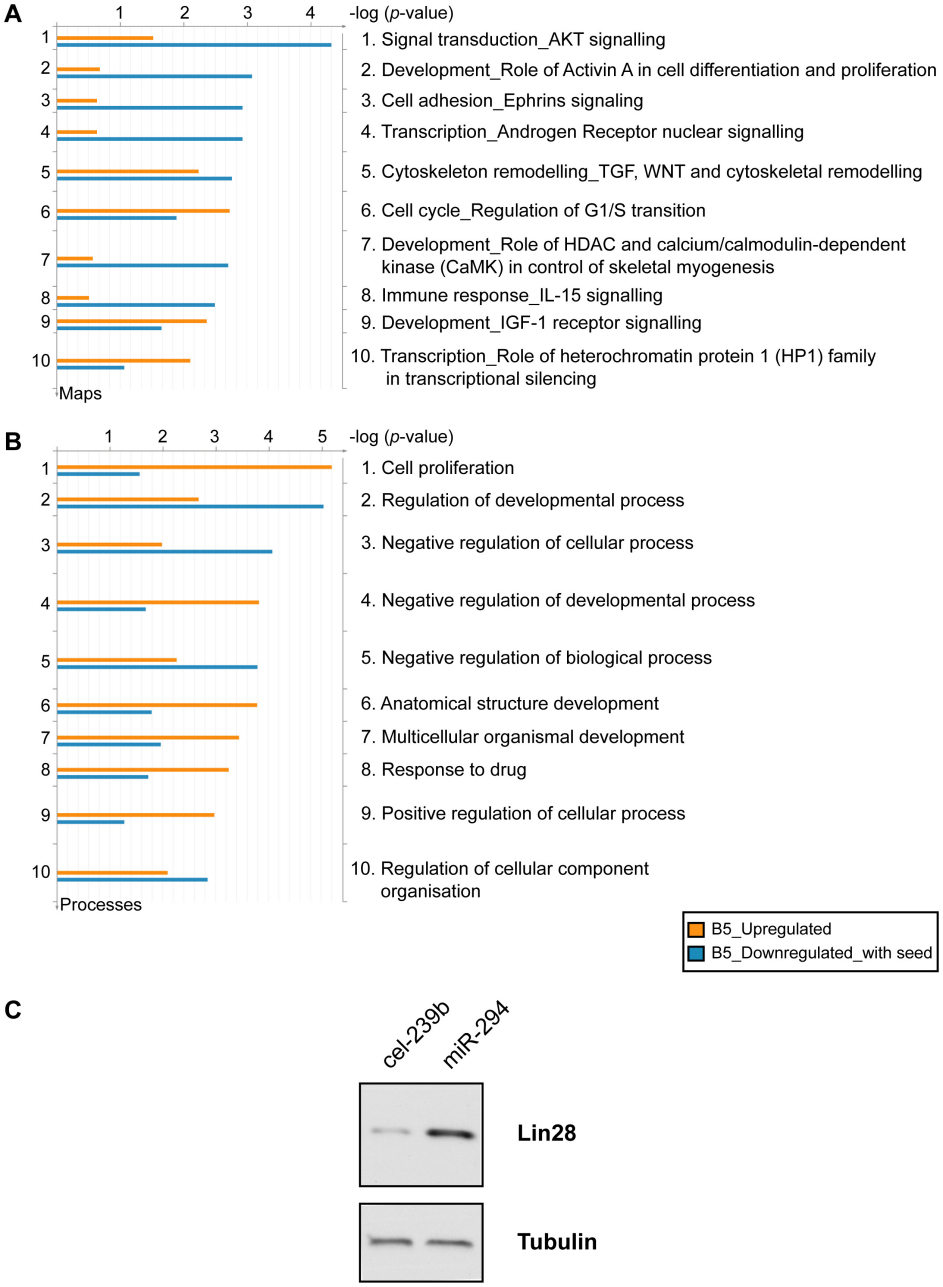
Functional enrichment of genes differentially expressed upon miR-294 transfection. Genes identified with differential expression from the microarray analysis were assigned functions based on Gene Ontology (GO) annotations using the GeneGo tool (GeneGo Inc). Functional ontologies are represented by (A) GeneGo canonical pathway maps (B) GO processes. The top ten terms are shown for downregulated genes (*B*-statistic >5) with at least one 6-mer site in its 3′ UTR (orange) and upregulated genes (*B*-statistic >5) (blue). (C) Western blot of Lin28 expression in *Dicer1* ^Δ/Δ^+Ago2-myc ES cells transfected with cel-239b control miRNA or miR-294.

To gain an insight into the biological role of miR-294, we performed a Gene Ontology (GO) analysis on the upregulated and downregulated genes (with at least one 6-mer in their 3′ UTR) selected from the microarray (*B*-statistic >5). The top ten terms included enrichment for genes involved in cell cycle (regulation of the G1-S transition), and development and transcription (Figure 5A). The miR-290-295 cluster has been described as a ‘Trojan horse’ inside ES cells to bring about differentiation [29]. If this were indeed the case, then one would expect to find an enrichment of differentiation-associated terms in the upregulated genes. This is not the case. Instead, the majority of terms associated with differentiation were more enriched in the downregulated genes. In addition, *Lin28* was upregulated by miR-294 transfection into *Dicer1* ^Δ/Δ^ *+*Ago2-myc ES cells (Figure 5C). *Lin28* is considered to be important for stem cell maintenance by blocking the processing of let-7 [30], [31], a critical miRNA involved in differentiation [32]. Furthermore, *Lin28*, in conjunction with *Nanog*, *Oct-4* and *Sox2* can reprogram human fibroblasts into pluripotent cells [33]. No pluripotency genes were detected amongst the downregulated genes. This is consistent with a potential role of miR-294 in the maintenance of the pluripotent state because co-expression of miRNAs that directly target the pluripotency factors would be detrimental to ES cells.

**Figure 6:**
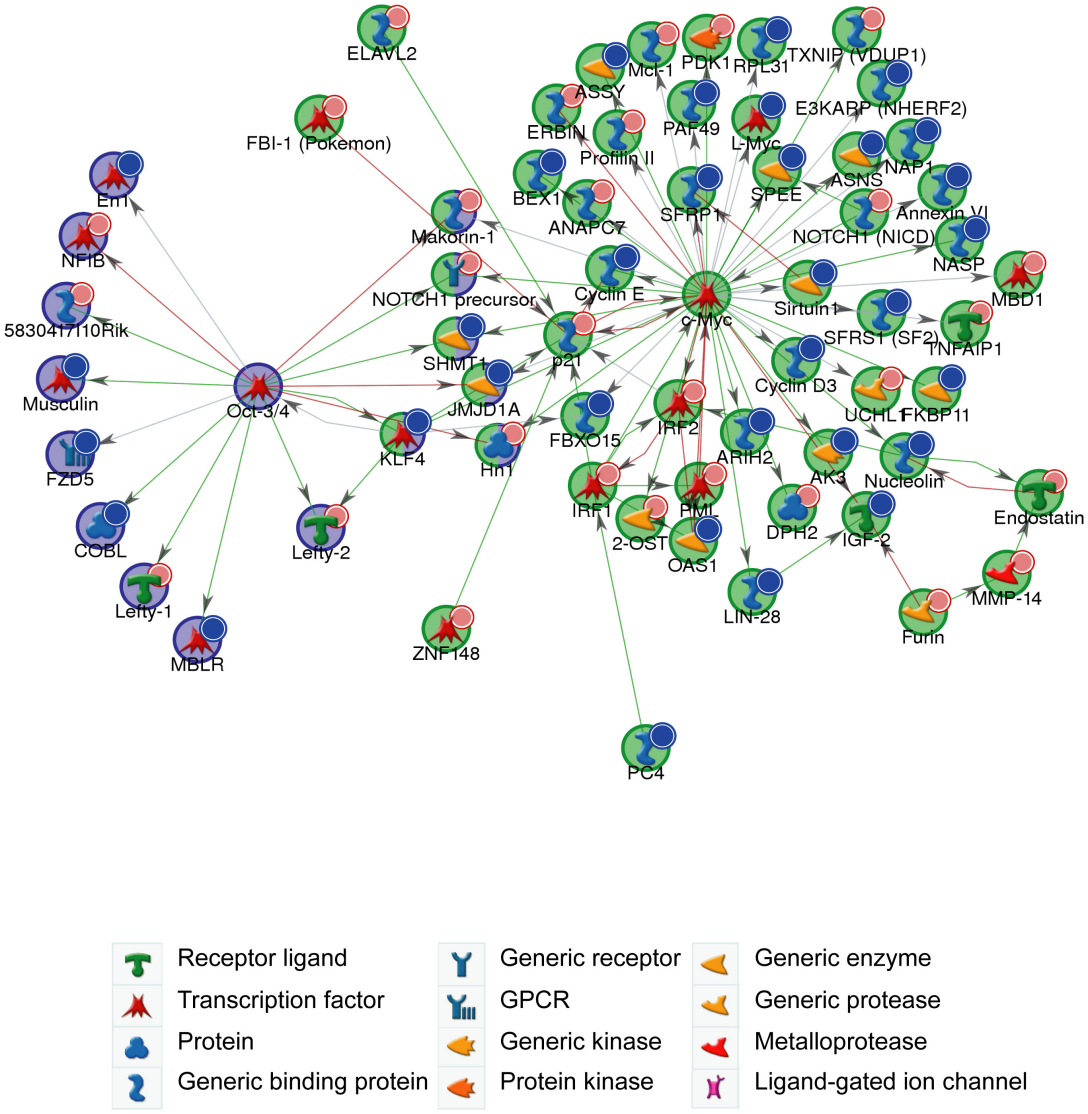
c-Myc and Oct-4 networks. GeneGo network generated from microarray gene expression data. Downregulated genes (*B*-statistic >5) with at least one 6-mer site in its 3′ UTR are marked with red circles; upregulated (*B*-statistic >5) with blue circles. c-Myc network (*p*-value <1.70×10^−130^); Oct-4 network (*p*-value <8.20×10^−35^). Green arrows indicate an activating effect; red arrows an inhibitory effect and grey is an unspecified effect.

In keeping with cell proliferation as the top-ranking functional GO category (Figure 5B), miR-294 has previously been reported to be able to substitute for *c-Myc*, but not *Oct-4*, *Sox2* or *Klf4*, in the reprogramming of mouse embryonic fibroblasts (MEFs) into induced Pluripotent Stem (iPS) cells [14]. The authors posited that miR-294 acts as a downstream target of *c-Myc*, which binds to the promoter region of *mir-290-295*. To test the alternative (and not exclusive) possibility that miR-294 may also substitute for *c-Myc* by regulating shared targets, a GeneGo (GeneGo Inc) network was generated using the downregulated and upregulated genes (with a *B*-statistic >5) from the microarray as an input list. The resulting networks revealed enrichment for a subset of targets of the c-Myc network (Figure 6). c-Myc was a central node with direct regulatory connections to target genes, but it was not itself a target of miR-294. However, many c-Myc target genes were downregulated directly or upregulated indirectly by miR-294. This suggests that miR-294 and c-Myc regulate an overlapping set of target genes, which is consistent with their comparable roles during iPS induction [14]. Thus, from the GeneGo network, miR-294 appears to act synergistically with c-Myc on a subset of targets. However, miR-294 may also repress a subset of targets that are induced by c-Myc. Since the reprogramming process is not identical when miR-294 is substituted for *c-Myc* [14], this might perhaps explain some of the observed differences between them during the derivation of iPS cells. This network motif, in which c-Myc both activates a target, and inhibits it via miR-294, is described as “incoherent feedforward” regulation [34]. Our network also includes a previously hypothesised incoherent feedforward loop involving *Oct-4*, miR-294 and *Lefty1/Lefty2* [35]. The functional significance of the incoherent feedforward loop has not been fully delineated but it can accelerate the response following a stimulus by decreasing the target concentration, fine tune steady state levels and buffer against perturbation [34]. In conclusion, miR-294 may promote pluripotency through regulating a shared subset of c-Myc target genes rather than simply being a downstream effector of c-Myc, and through the upregulation of pluripotency-associated genes, such as *Lin28*.

## Discussion

Our findings show that miR-294 regulates a subset of c-Myc target genes, and upregulates Lin28. We did not observe substantial overlap between *Oct-4*, *Nanog* or *Sox2* regulated genes and miR-294-regulated transcripts. Thus, the effects on c-Myc and Lin28 are distinct from the other core pluripotency factors, consistent with previous data that proposed functional differences between targets of Myc and that of the other core pluripotency factors [36]–[38]. Furthermore, our conclusions are supported by a recent study [39]. However, we did not detect upregulation of *c-Myc* mRNA by miR-294 in our microarray results, but rather *Mycl1*, which belongs to the Myc family of transcription factors. This difference could be a result of the different microarray platforms used for the transcriptome analyses.

Indeed, we identified other networks that were enriched for miR-294-regulated transcripts, which included *Sp1*, *Esr1*, *Hnf4-alpha*, *p53* and *Stat3*. The integration of miRNAs into transcriptional networks offers versatility to the regulatory outputs: it enables miRNAs to affect the dynamical properties of transcription factor targets, modulate the strength of transcription factors, and increase the robustness of transcription factor networks. With respect to c-Myc, this model may provide insights on how the initial proliferation defects of *Dicer1*-null ES cells can be compensated with time [7], [8], and indicates a resetting of the transcriptional network in the absence of miRNAs.

This is the first study to use *Dicer1*-null ES cells combined with a biochemical approach to identify targets of a single miRNA at a time. A surprising finding was that there was a GC bias in hexamer composition in the 3′ UTRs of enriched transcripts. This effect was much greater for samples transfected with miR-294 than with cel-239b, implying that there is a biological basis for this association. Such transcripts with no predicted canonical seed matches may reflect binding to miRNAs that don't follow the rules of seed-driven target recognition or non-specific associations. Recently, a role for seed sequence-independent regulation has emerged for miR-328, which acts through its C-rich region to titrate a translational inhibitor, hnRNP E2 protein, from its target mRNA [40]. However, the possibility of non-canonical targets would require careful verification.

A pertinent issue for the biochemical approach is the ability of miRNAs to induce mRNA destabilisation independently of Ago2-catalysed slicing, as this could hamper the isolation of mRNAs that are being degraded. One possible way to address this in future work is to explore different Ago2 mutants that cannot interact with factors that bring about mRNA destabilisation, such as GW182 [41]. The use of such a mutant might improve the likelihood of retaining intact mRNAs and increase the efficiency of the pull down.

Despite a large discrepancy between miRNAs found in human and mouse ES cells, there are homologs of the miR-290-295 cluster in human ES cells (miR-371, miR-372 and miR-373) [10], alluding to an important role of these miRNAs in the pluripotency of mammalian ES cells. Given the expression of miR-294 in primordial germ cells [42] and its function in pluripotency, it will be interesting to determine the effects of miR-290-295 conditional deletion upon the germline.

## Materials and Methods

### Cell culture

Mice carrying floxed alleles of *Ago2* or *Dicer1* were described previously [43], [44]. *Ago2* ^flox/flox^;*Dicer1* ^flox/flox^ ES cells were derived from *Ago2* ^flox/flox^;*Dicer1* ^flox/flox^ blastocysts and cultured on feeders in media supplemented with leukaemia inhibitory factor. *Ago2* ^flox/flox^;*Dicer1* ^flox/flox^ ES cells were transfected with linearised plasmids of pCAG-Ago2-myc, pCAG-Ago2-myc-MUT or pCAG-myc, and selected with G418. To excise the floxed alleles of *Ago2* and *Dicer1* in *Ago2* ^flox/flox^;*Dicer1* ^flox/flox^ stable clones, ES cells were transfected with Cre-GFP plasmid (Addgene). After 24 hr, GFP-positive cells were flow-sorted (FACSAria, BD Biosciences). 2000 or 4000 cells/10 cm dish were plated to enable picking of single colonies. The genotype of individual clones was determined by PCR using primers: Dicer-FN 5′-GGT TAC ATG GCT AGA CTC AAA GC-3′; Dicer-RN 5′-AGG TGC CTT TCG TTT AGG AAC-3′; Dicer-FWF 5′-AAA GCA GAA CTC TAA TGC CCC-3′. It was further confirmed by profiling the expression of mature miRNAs from the miR-290-295 cluster (Figure S1C). ES cells were maintained in ES cell medium, in the absence of feeders on gelatinised tissue culture plates. ES medium consisted of:: DMEM/F12 (Invitrogen), 15% FCS (Gibco), 2 mM L-glutamine (Gibco), 0.1 mM non-essential amino acids (Gibco), 1 mM sodium pyruvate (Sigma), 0.12% sodium bicarbonate (Sigma), 10 µM β-mercaptoethanol (Gibco), 50 µg/ml penicillin/streptomycin (Gibco), and 2×10^3^ U/ml LIF (Chemicon).

### Plasmids

The *Ago2* coding sequence was amplified from 129Sv/Ev mouse ES cell cDNA by PCR using primers: Ago2-5′ 5′- AGA ATT CAT GTA CTC GGG AGC CGG CCC CGT TCT T-3′; Ago2-3′ 5′-ATG CGG CCG CTC ACA GAT CCT CTT CTG AGA TGA GTT TTT GTT CAG CAA AGT ACA TGG TGC GCA G-3′ to contain a carboxy-terminal myc-epitope tag. Q633R and H634A point-mutations were introduced by site-directed mutagenesis using the QuiKChange kit (Stratagene). For the mammalian expression constructs, wild-type and point-mutated *Ago2-*myc in pCAGIG were digested with Sal1-Not1 to include the CAG promoter upstream of the *Ago2-myc* coding sequence and ligated into pEGFP-1 (Clontech), which was pre-digested with Sal1-Not1 to remove the EGFP coding sequence.

### Transfection

Transfections were performed using the Mouse ES Cell Nucleofection Kit (Lonza), and program A23 of Nucleofactor I apparatus (Lonza), as specified by the manufacturer's instructions. Transfections of miRNA mimics mmu-miR-294 and cel-239b (Dharmacon) was performed as described in [17]. Briefly, for microarray analysis, approximately 4×10^6^ *Dicer1*-null ES cells were resuspended in 90 µl mouse ES cell Nucleofection Solution. 300 pmol of miR-294 and 1 µg pEGFP-1 (Clontech) plasmid (which served as a control for transfection efficiency) were diluted in 10 µl Nucleofection Solution, mixed with the cells and transferred to a Lonza cuvette. For RNA-IP experiments, cells were transfected with 300 pmol of miR-294 or cel-239b control miRNA (Dharmacon) and harvested 12–16 hr after transfection for immunoprecipitation.

### Western blotting

Cells were lysed in lysis buffer (50 mM Tris-HCl pH 8.0, 150 mM NaCl, 1% NP-40, 0.5% sodium deoxycholate, 0.1% SDS, and protease inhibitors). Equal concentration of the lysed proteins were separated on polyacrylamide-SDS gels, transferred onto PVDF Hybond membrane and probed with the following primary antibodies: anti-Ago2 (Abnova, 1∶1000), Lin28 (Cell Signaling Technology, 1∶1000), Tubulin (Sigma, 1∶5000). This was followed by incubation with horseradish peroxidase-conjugated secondary antibodies. ECL kits (Amersham) were used for detection.

### Immunofluorescence

Cells were grown on *Lab-Tek* chamber slides and fixed with 4% paraformaldehyde in PBS for 15 minutes at room temperature. Cells were blocked in 1% BSA/0.1% Triton X-100 in PBS and incubated O/N at 4°C with the following primary antibodies: anti-Eomes (eBioscience, 1∶200), anti-Oct3/4 (BDBiosciences, 1∶200). This was followed by incubation with AlexaFluor secondary antibodies (1∶500; Molecular Probes) and DAPI (1∶1000; Sigma), for 1 hr at room temperature.

### miRNA– and RNA–immunoprecipitation

miRNA- and RNA-immunoprecipitation was performed in native conditions as described [45]–[47]. 2.5×10^7^ cells were pelleted and resuspended in 220 µl ice-cold Polysomal Lysis Buffer [100 mM KCl, 5 mM MgCl_2_, 10 mM HEPES (pH 7.0; Gibco), 0.5% NP40, 1 mM DTT, 200 U Recombinant RNasin Ribonuclease Inhibitor (Promega), 200 U SUPERase•In RNase inhibitor, and Protease Inhibitor Cocktail]. The lysate was passed through a 27G needle five times, incubated on ice for 30 min at 4°C and then transferred to –80°C to promote lysis. The lysate was then thawed on ice, centrifuged (15 min, maximum speed, 4°C), and the supernatant was transferred to a new tube. 400 U DNase (Roche) was added and incubated on 30 min at 4°C. 20 µl of the lysate was saved for the INPUT at –80°C. The remaining lysate was diluted in 800 µl NT2 buffer [50 mM Tris-Hcl (pH 7.4), 150 mM NaCl, 1 mM MgCl_2_, 0.05% NP40, Protease Inhibitor Cocktail, 200 U Recombinant RNasin Ribonuclease Inhibitor] and pre-cleared for 2 hr at 4°C with 20 µl Dynabeads-Protein A (Invitrogen), which had been pre-blocked in 0.5% BSA +1 mg/ml yeast tRNA (Ambion). The supernatant was incubated with 20 µl anti-myc (Cell Signaling Technology) antibody O/N at 4°C and 200 U Recombinant RNasin Ribonuclease Inhibitor. The next day, the RNA/antibody complex was precipitated by addition of 50 µl Dynabeads-Protein A for 2 hr at 4°C. The beads were washed with 1 ml NT2 buffer four times and then resuspended in 100 µl NT2 buffer and transferred to a fresh tube. 80 µl of NT2 buffer was added to 20 µl of the stored INPUT. RNA was extracted with 1 ml Trizol according to the manufacturer's protocol and RNA was resuspended in 20 µl H_2_O. To amplify mRNA 20 cycles for sequencing, 1 µl of IP (10–40 ng/µl) and 1 µl of INPUT (diluted to 0.5–2.5 ng/µl) were used. For sequencing of mRNAs, the single cell method was modified according to [48]. For 220-plex microRNA expression profiling, 220 miRNAs were reverse transcribed, amplified and then analysed by Q-PCR, as described [49], [50].

### Real-time quantitative PCR

Quantitative PCR was performed on an ABI PRISM 7000 sequence detection system (Applied Biosystems). For SYBR Green fluorescent nucleic acid dye, primers were designed in order to achieve product lengths between 50 and 100 bp. TaqMan probes were used for detection of mature miRNAs by Q-PCR. The reaction conditions were: 95°C 10 min, 40× (95°C 15 s, 60°C 1 min). Data was normalised to *HPRT*, or INPUT RNA amount in the case of RNA- and miRNA-immunoprecipitation experiments, using the ΔΔC_T_ method [51].

### Microarray

Feeder-free *Ago2^Δ^* ^/*Δ*^;*Dicer1^Δ^* ^/*Δ*^+Ago2*-*myc ES cells were transfected in triplicate with 1 µg of pEGFP-1 and 300 pmol of miR-294 mimic or *C. elegans* cel-239b control miRNA and harvested 24 hr later. Cells were sorted for GFP expression and total RNA was isolated using the RNeasy Mini kit (Qiagen). 1 µg of total RNA from each triplicate (six samples in total) was sent to Cambridge Genomic Services, Cambridge University for sample processing and hybridisation to MouseWG-6 v2.0 Illumina microarrays. Cambridge Genomic Services performed the quality control analysis, gene selection, and data normalisation. Microarray data analysis was carried out using the R language with Bioconductor packages [52] and custom-written code. GO and network analysis were performed using the online software MetaCore from GeneGo Inc. Microarray data were deposited in the Gene Expression Omnibus (GEO) database (Accession Code: GSE20048).

### RNA–Sequencing

The amplified cDNAs from the IP and INPUT were subjected to SOLiD library preparation by ABI. The cDNAs were then sequenced by ABI's next-generation sequencing SOLiD system. RNA-Sequencing reads were mapped to RefSeq transcripts using ABI's pipeline, as described [48]. To visualise the data, we typically plotted the fraction of reads for a given gene which came from the IP library: 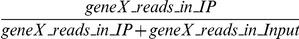



Differential representation was measured as the probability that 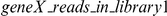 was drawn from a binomial distribution with
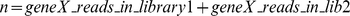
and
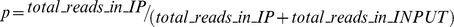



Sequencing data were deposited in the GEO database (Accession Code: GSE20199).

## Supporting Information

Figure S1 *Dicer1*-null ES cells. (A) Genomic organisation of the *mir-290-295* cluster of miRNAs from mouse chromosome 7. Expression profiling of miR-290-295 mature miRNAs in *Ago2* ^flox/flox^;*Dicer1* ^flox/flox^ ES cells. Error bars indicate S.D. (B) *Ago2* ^flox/flox^;*Dicer1* ^flox/flox^ and *Ago2* ^Δ/Δ^;*Dicer1* ^Δ/Δ^+Ago2-myc ES cells were immunostained for the pluripotency marker Oct-4 (red), and the trophoblast marker Eomes (green). Trophoblast stem (TS) cells were used as a negative control for Oct-4 and a positive control for Eomes. Scale bar: 10 µm. (C) Functional loss of *Dicer1* was confirmed by profiling the mature miRNAs from the miR-290-295 cluster in *Ago2* ^Δ/Δ^;*Dicer1* ^Δ/Δ^+Ago2-myc ES cells. Values calculated for *Ago2* ^flox/flox^;*Dicer1* ^flox/flox^ were set as one. Error bars indicate S.D.
(3.01 MB TIF)

Figure S2 RNA-Immunoprecipitation of Ago2-myc. (A) Immunoprecipitation of wild-type (WT) or catalytically-inactive (MUT) myc-tagged Ago2 from *Dicer1* ^Δ/Δ^+Ago2-myc transgenic ES cells using anti-myc antibody. The Western blot was immunoblotted with anti-Ago2 antibody (SUP: supernatant). (B) Isolation of Ago2-myc bound RNA from *Ago2* ^flox/flox^;*Dicer1* ^flox/flox^ transgenic ES cells and miRNA profiling of miR-292-3p, miR-293 and miR-294. Error bars indicate S.D. Data is normalised to levels of miRNA in the INPUT and are relative to the myc tag control. (C) Isolation of Ago2-myc bound RNA from *Dicer1* ^Δ/Δ^+Ago2-myc ES cells transfected with miR-294 or cel-239b, followed by Q-PCR of a known miR-294 target, *Cdkn1a*. Error bars indicate S.D. Data is normalised to levels of transcript in the INPUT, and are relative to cel-239b-transfected cells.
(1.55 MB TIF)

Figure S3 Experimental scheme for RNA-IP strategy. (A) Scheme of RNA-IP strategy. miR-294 or cel-239b was transfected into *Dicer1* ^Δ/Δ^+Ago2-myc ES cells. Cells were harvested 12 hr post-transfection and lysed. A fraction of the cell lysate was saved for the INPUT. Anti-myc antibodies were used to immunoprecipitate Ago2-myc. RNA was then isolated from immunoprecipitated Ago2-myc and the INPUT, and then reverse transcribed. cDNAs were amplified, subjected to library preparation and sequenced. (B) Transfection efficiency of small RNAs in unsorted *Dicer1* ^Δ/Δ^+Ago2-myc-WT ES cells transfected with different concentrations of miR-291a-5p and harvested 12-16 hr post-transfection. Data is normalised to *Hprt* and is relative to *Dicer1* ^Δ/Δ^+Ago2-myc-WT ES cells, which serves as a control. Endogenous miR-291a-5p levels in *Ago2* ^flox/flox^;*Dicer1* ^flox/flox^ ES cells are included as a comparison for transfected levels of miR-291a-5p. Error bars indicate S.D.
(0.58 MB TIF)

Figure S4 Cumulative histogram for IP enrichment. The *x*-axis represents IP/(INPUT + IP). Black line: Ago2-myc transfected with cel-239b. Blue line: Ago2-myc transfected with miR-294. Red line: Ago2-myc-MUT transfected with miR-294. Values close to 0.5 indicate that the IP and INPUT libraries are similar whereas values above 0.5 suggest that there is extra enrichment in the IP.
(0.45 MB TIF)

Table S1 Genes upregulated on the microarray with a *B*-statistic >5. A negative log FC (Fold Change) indicates upregulation.
(0.05 MB DOC)

Table S2 Sylamer list of microarray target predictions. The number of 6-mer, 7-mer and 8-mer in their 3′ UTRs are indicated. The top 127 downregulated genes with at least one miR-294 seed match and a *B*-statistic >5 are highlighted in bold typeface.
(0.18 MB DOC)
