# Supplementary figures and images for: Genome-Wide Identification of Targets and Function of Individual MicroRNAs in Mouse Embryonic Stem Cells
Source: PLoS Genet. 2010 Oct 21;6(10):e1001163. doi: 10.1371/journal.pgen.1001163 (PMC2958809; doi:10.1371/journal.pgen.1001163)

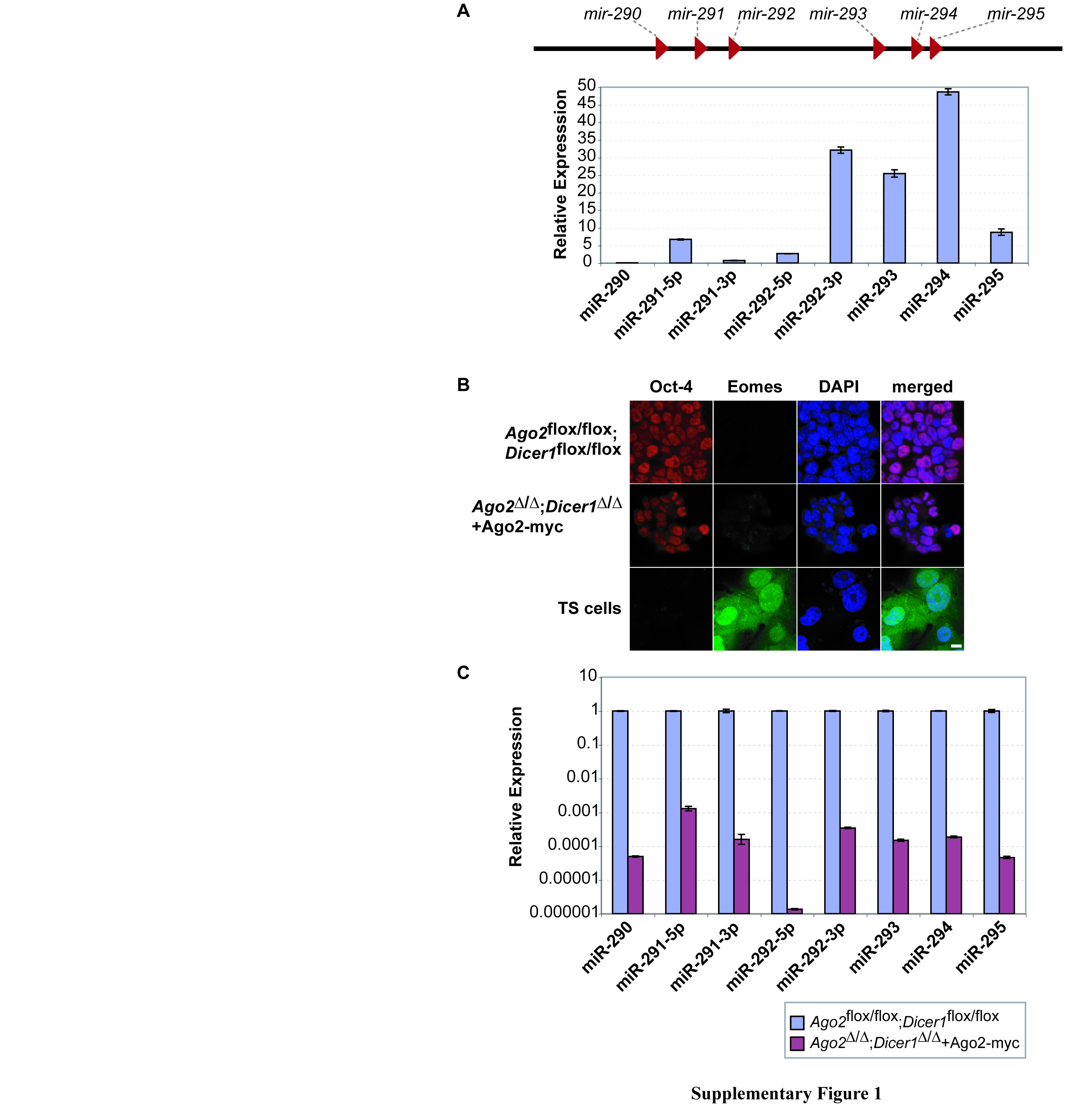

Supplement: Figure S1 — Dicer1-null ES cells. (A) Genomic organisation of the mir-290-295 cluster of miRNAs from mouse chromosome 7. Expression profiling of miR-290-295 mature miRNAs in Ago2 flox/flox;Dicer1 flox/flox ES cells. Error bars indicate S.D. (B) Ago2 flox/flox;Dicer1 flox/flox and Ago2 Δ/Δ;Dicer1 Δ/Δ+Ago2-myc ES cells were immunostained for the pluripotency marker Oct-4 (red), and the trophoblast marker Eomes (green). Trophoblast stem (TS) cells were used as a negative control for Oct-4 and a positive control for Eomes. Scale bar: 10 µm. (C) Functional loss of Dicer1 was confirmed by profiling the mature miRNAs from the miR-290-295 cluster in Ago2 Δ/Δ;Dicer1 Δ/Δ+Ago2-myc ES cells. Values calculated for Ago2 flox/flox;Dicer1 flox/flox were set as one. Error bars indicate S.D. (3.01 MB TIF) [file pgen.1001163.s001.tif]

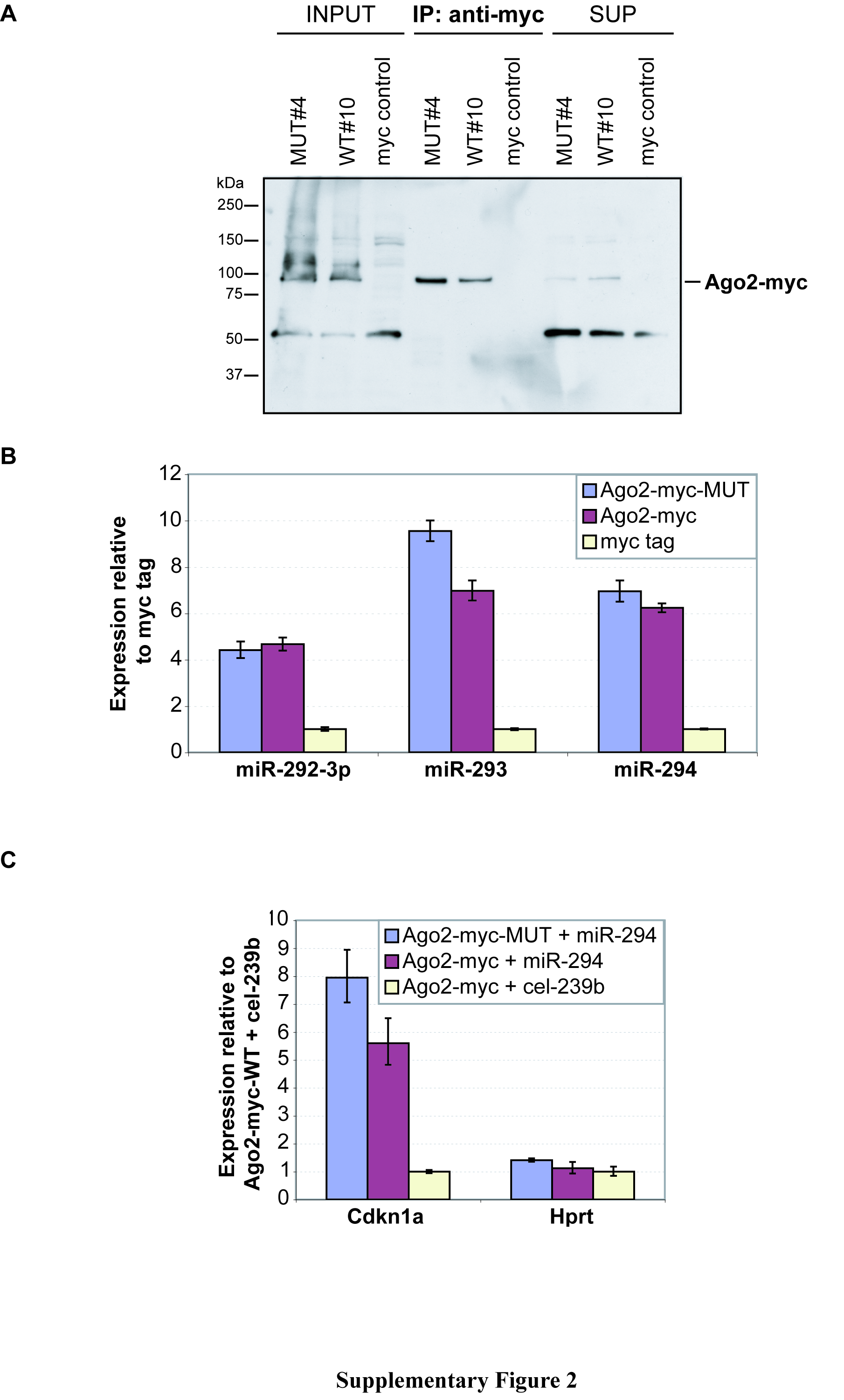

Supplement: Figure S2 — RNA-Immunoprecipitation of Ago2-myc. (A) Immunoprecipitation of wild-type (WT) or catalytically-inactive (MUT) myc-tagged Ago2 from Dicer1 Δ/Δ+Ago2-myc transgenic ES cells using anti-myc antibody. The Western blot was immunoblotted with anti-Ago2 antibody (SUP: supernatant). (B) Isolation of Ago2-myc bound RNA from Ago2 flox/flox;Dicer1 flox/flox transgenic ES cells and miRNA profiling of miR-292-3p, miR-293 and miR-294. Error bars indicate S.D. Data is normalised to levels of miRNA in the INPUT and are relative to the myc tag control. (C) Isolation of Ago2-myc bound RNA from Dicer1 Δ/Δ+Ago2-myc ES cells transfected with miR-294 or cel-239b, followed by Q-PCR of a known miR-294 target, Cdkn1a. Error bars indicate S.D. Data is normalised to levels of transcript in the INPUT, and are relative to cel-239b-transfected cells. (1.55 MB TIF) [file pgen.1001163.s002.tif]

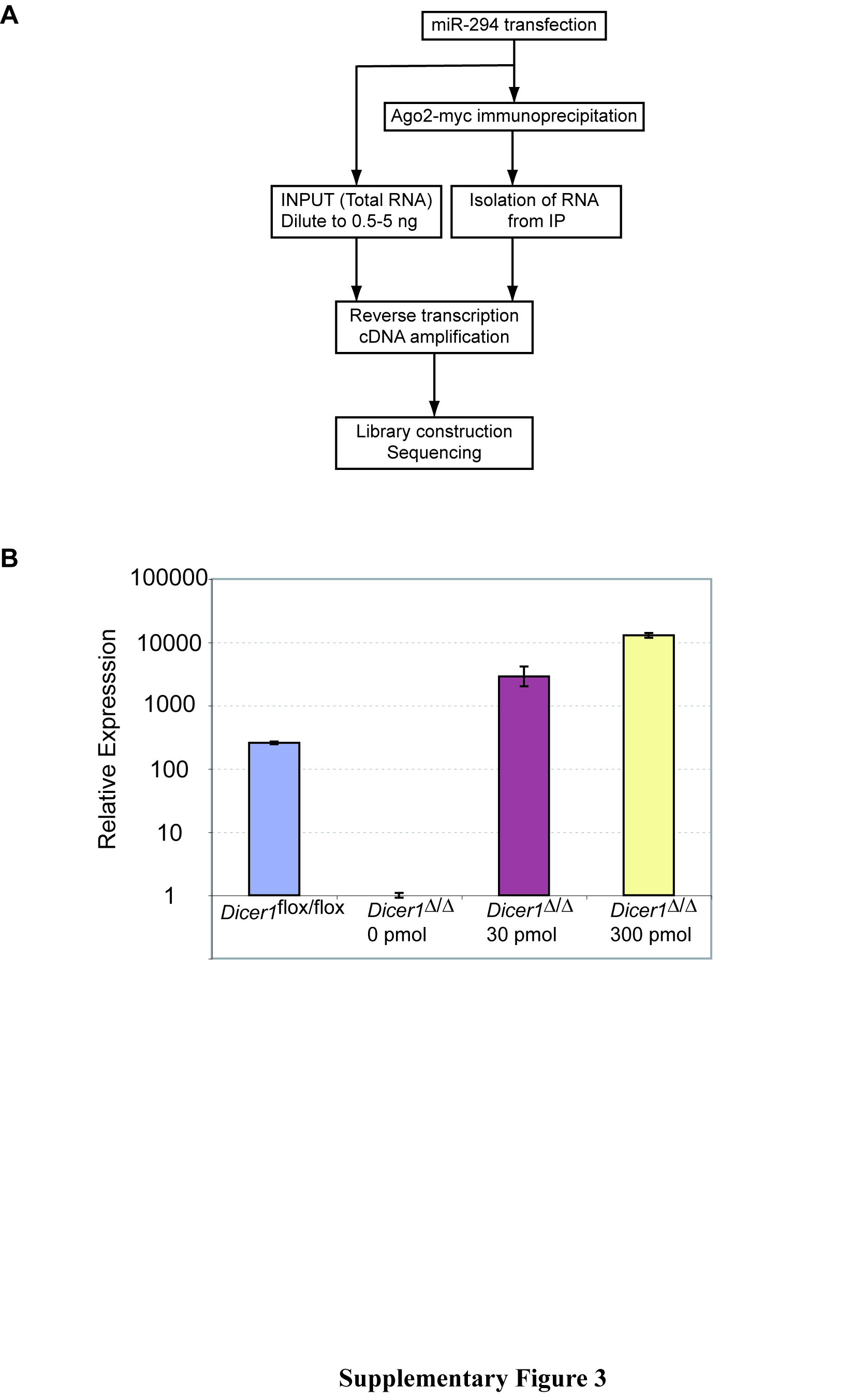

Supplement: Figure S3 — Experimental scheme for RNA-IP strategy. (A) Scheme of RNA-IP strategy. miR-294 or cel-239b was transfected into Dicer1 Δ/Δ+Ago2-myc ES cells. Cells were harvested 12 hr post-transfection and lysed. A fraction of the cell lysate was saved for the INPUT. Anti-myc antibodies were used to immunoprecipitate Ago2-myc. RNA was then isolated from immunoprecipitated Ago2-myc and the INPUT, and then reverse transcribed. cDNAs were amplified, subjected to library preparation and sequenced. (B) Transfection efficiency of small RNAs in unsorted Dicer1 Δ/Δ+Ago2-myc-WT ES cells transfected with different concentrations of miR-291a-5p and harvested 12-16 hr post-transfection. Data is normalised to Hprt and is relative to Dicer1 Δ/Δ+Ago2-myc-WT ES cells, which serves as a control. Endogenous miR-291a-5p levels in Ago2 flox/flox;Dicer1 flox/flox ES cells are included as a comparison for transfected levels of miR-291a-5p. Error bars indicate S.D. (0.58 MB TIF) [file pgen.1001163.s003.tif]

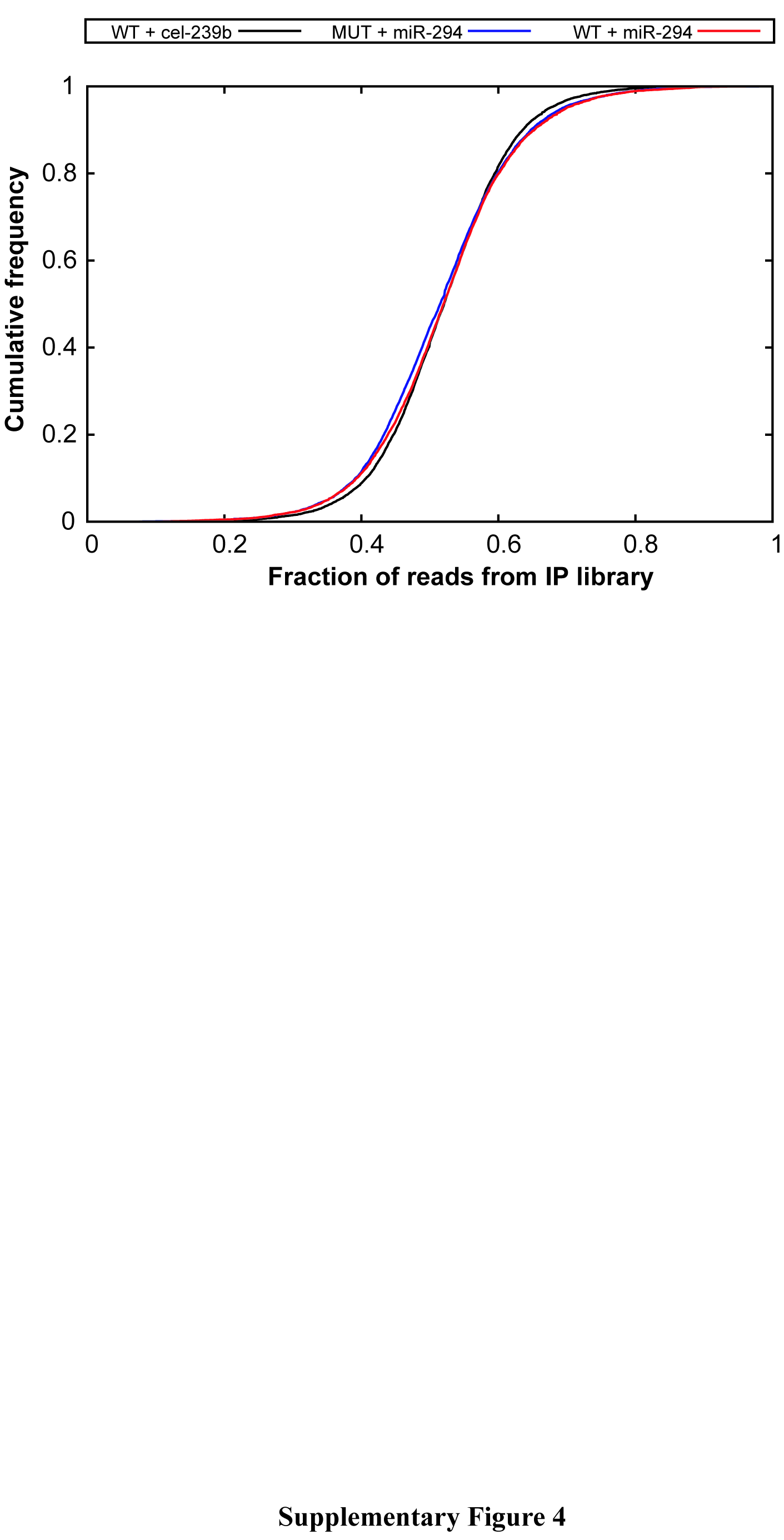

Supplement: Figure S4 — Cumulative histogram for IP enrichment. The x-axis represents IP/(INPUT + IP). Black line: Ago2-myc transfected with cel-239b. Blue line: Ago2-myc transfected with miR-294. Red line: Ago2-myc-MUT transfected with miR-294. Values close to 0.5 indicate that the IP and INPUT libraries are similar whereas values above 0.5 suggest that there is extra enrichment in the IP. (0.45 MB TIF) [file pgen.1001163.s004.tif]
