# Supplementary material for: Genome-Wide Identification of Targets and Function of Individual MicroRNAs in Mouse Embryonic Stem Cells
Source: PLoS Genet. 2010 Oct 21;6(10):e1001163. doi: 10.1371/journal.pgen.1001163 (PMC2958809; doi:10.1371/journal.pgen.1001163)
Supplement: Table S1 — Genes upregulated on the microarray with a B-statistic >5. A negative log FC (Fold Change) indicates upregulation. (0.05 MB DOC) [file pgen.1001163.s005.doc]

**Supplementary Table 1** **Genes upregulated on the microarray with a *B*-statistic > 5.** A negative log FC (Fold Change) indicates upregulation.

| Gene | logFC |
| --- | --- |
| LOC100046049 | -2.109686845 |
| Spink3 | -1.621520596 |
| Lin28 | -1.515395928 |
| Gprasp1 | -1.007704863 |
| Fkbp11 | -0.946955754 |
| Kit | -1.069216676 |
| Rangrf | -0.907842718 |
| Adcy2 | -0.770518805 |
| Igfbp2 | -0.689317484 |
| Lin28 | -1.408166329 |
| LOC100045304 | -0.693956232 |
| Anxa6 | -0.816285607 |
| Gprasp2 | -0.646164035 |
| Myo1f | -0.594643666 |
| Bex2 | -0.685552384 |
| Sfrp1 | -0.721537813 |
| Gdf1 | -0.549757247 |
| Reep1 | -0.604965792 |
| Gp38 | -0.643284318 |
| 6720458D17Rik | -0.574333631 |
| Eras | -0.751954695 |
| Shmt1 | -0.598102456 |
| Fgf17 | -1.04866358 |
| Calca | -0.560237978 |
| Serpinb6c | -0.64704685 |
| Slc9a3r2 | -0.877755208 |
| Gtsf1l | -0.600429729 |
| Ass1 | -0.689779001 |
| Smtnl2 | -0.577237556 |
| Klf4 | -0.65458974 |
| Bex2 | -0.750785743 |
| Sema4a | -0.563622658 |
| Pwp2 | -0.53661323 |
| Slc25a15 | -0.508103759 |
| Igfbp2 | -0.605372612 |
| Gm428 | -0.887278553 |
| D10Ertd610e | -0.495719141 |
| Slc25a20 | -0.56316055 |
| Osta | -0.521209396 |
| LOC548597 | -0.569997888 |
| Lsm6 | -0.473517738 |
| Srm | -0.587043215 |
| Ly6g6e | -0.606720966 |
| Oas1g | -0.712236459 |
| BC028528 | -0.531123619 |
| LOC383897 | -0.836170723 |
| Dnajc6 | -0.525592222 |
| 2410116G06Rik | -0.58638268 |
| B3gnt5 | -0.509210754 |
| OTTMUSG00000001305 | -0.440202989 |
| E130012A19Rik | -0.657943446 |
| Raet1b | -0.459671349 |
| Mycl1 | -0.56278244 |
| Ncl | -0.513959558 |
| BC099439 | -0.439159064 |
| LOC235857 | -0.544035902 |
| Arih2 | -0.594658939 |
| Etsrp71 | -0.500019032 |
| Cdc5l | -0.531031097 |
| Pnma2 | -0.58048002 |
| Jam2 | -0.505210424 |
| Apoa2 | -0.548083034 |
| Cobl | -0.586543544 |
| Msc | -0.527283259 |
| Pla2g1b | -0.494187258 |
| Klk8 | -0.498550872 |
| Gprasp1 | -0.631772956 |
| Tcfl5 | -0.462536639 |
| Ccdc43 | -0.45929912 |
| Klhl22 | -0.526586092 |
| X99384 | -0.55933609 |
| Gsta3 | -0.540896533 |
| Ung | -0.685611395 |
| Fst | -0.532680486 |
| 1700019N12Rik | -0.426056459 |
| 4931407G18Rik | -0.409977792 |
| Sfrp1 | -0.630079617 |
| Pabpc4 | -0.450485626 |
| 1810015A11Rik | -0.389900455 |
| Pqlc1 | -0.53628953 |
| LOC433722 | -0.466995191 |
| Fgf17 | -0.457786925 |
| Rpp25 | -0.602974646 |
| Sfrs1 | -0.472004971 |
| Tmc6 | -0.411668215 |
| 5630401D24Rik | -0.382507418 |
| Ccnh | -0.577188879 |
| 2900062L11Rik | -0.526327239 |
| 1190003J15Rik | -0.525148102 |
| Wdr61 | -0.379111326 |
| Kcnk5 | -0.520868019 |
| Glipr2 | -0.39714022 |
| Gtpbp4 | -0.438163352 |
| Cpxm1 | -0.596971339 |
| Fbxo15 | -0.61566838 |
| Atp2a3 | -0.377544349 |
| D6Wsu163e | -0.371232191 |
| Drg2 | -0.403046687 |
| Asns | -0.402891749 |
| Mylpf | -0.399173433 |
| Dkk3 | -0.362249854 |
| Ube2cbp | -0.433947757 |
| Mcm10 | -0.401390592 |
| Ccne1 | -0.474160879 |
| Dnajc6 | -0.505169974 |
| Pcyt1b | -0.417338902 |
| Klf4 | -0.529624818 |
| Mybl2 | -0.360483624 |
| Mybl2 | -0.502217998 |
| C130035G06Rik | -0.703021033 |
| Plac8 | -0.351126058 |
| LOC100047579 | -0.473904034 |
| Rpo2tc1 | -0.346067084 |
| Nasp | -0.347932353 |
| Calca | -0.372684884 |
| Isy1 | -0.354343243 |
| Anp32a | -0.360677886 |
| LOC329984 | -0.424741132 |
| Pus10 | -0.370948877 |
| Psip1 | -0.429345997 |
| Smarca1 | -0.412447517 |
| Paox | -0.431832732 |
| BC028528 | -0.339606205 |
| Bex4 | -0.569487626 |
| Zfp473 | -0.395499648 |
| Cdv3 | -0.339528759 |
| LOC546233 | -0.448537025 |
| 1190017O12Rik | -0.401695977 |
| Suhw2 | -0.332861317 |
| Rfx2 | -0.39522445 |
| Alpk3 | -0.392680699 |
| Rab4a | -0.580283813 |
| ENSMUSG00000068790 | -0.358881964 |
| Cxcl12 | -0.44473122 |
| Nap1l1 | -0.455719663 |
| OTTMUSG00000010673 | -0.465456106 |
| Ccnd3 | -0.476216246 |
| Jmjd1a | -0.365732631 |
| Rpl31 | -0.41386483 |
| Upp1 | -0.392102203 |
| Txnl4a | -0.34325156 |
| Timm8a1 | -0.35350047 |
| Nt5dc2 | -0.399026481 |
| Upp1 | -0.337029767 |
| Apoa2 | -0.516762899 |
| AI850995 | -0.440506226 |
| Tubb2b | -0.451162097 |
| Ndp52 | -0.649618079 |
| 1190028F09 | -0.354412462 |
| En1 | -0.4041423 |
| Lck | -0.371350617 |
| Seh1l | -0.366755669 |
| Nip7 | -0.473800645 |
| Pcgf6 | -0.395029763 |
| Akp2 | -0.505671645 |
| D0HXS9928E | -0.378947431 |
| Gpr83 | -0.343061965 |
| Fzd5 | -0.448789284 |
| 2900060P06Rik | -0.339532837 |
| Rasl11b | -0.366878845 |
| Nol8 | -0.367695946 |
| LOC433721 | -0.515254378 |
| 1300013J15Rik | -0.357549932 |
| Atp2a3 | -0.310697555 |
| Lgtn | -0.39102621 |
| Grb10 | -0.523310645 |
| Zfp473 | -0.362440154 |
| Srm | -0.583126503 |
| Gps1 | -0.646945032 |
| Axin2 | -0.388902652 |
| Ak3l1 | -0.541891684 |
| Tmc6 | -0.383306022 |
| Ly6g6e | -0.395835284 |
| Tubb2b | -0.396377921 |
| Igf2 | -0.470170443 |
| Armcx2 | -0.488889617 |
| Sirt1 | -0.449654302 |
| Dkk3 | -0.315214354 |
| LOC380927 | -0.499610373 |
| Idh1 | -0.36235217 |
| Pja1 | -0.383299259 |
| Eef1d | -0.356784562 |
