# Supplementary material for: Genome-Wide Identification of Targets and Function of Individual MicroRNAs in Mouse Embryonic Stem Cells
Source: PLoS Genet. 2010 Oct 21;6(10):e1001163. doi: 10.1371/journal.pgen.1001163 (PMC2958809; doi:10.1371/journal.pgen.1001163)
Supplement: Table S2 — Sylamer list of microarray target predictions. The number of 6-mer, 7-mer and 8-mer in their 3′ UTRs are indicated. The top 127 downregulated genes with at least one miR-294 seed match and a B-statistic >5 are highlighted in bold typeface. (0.18 MB DOC) [file pgen.1001163.s006.doc]

**Supplementary Table 2 Sylamer list of microarray target predictions.**

The number of 6-mer, 7-mer and 8-mer in their 3’ UTRs are indicated. The top 127 downregulated genes with at least one miR-294 seed match and a *B*-statistic > 5 are highlighted in bold typeface.

| Gene | GCACTT | AGCACTT | AGCACTTT |
| --- | --- | --- | --- |
| **Lefty1** | 0 | 1 | 0 |
| **Casp2** | 3 | 1 | 0 |
| **Syde1** | 0 | 1 | 0 |
| **Cdkn1a** | 1 | 1 | 1 |
| **Mbd1** | 1 | 1 | 0 |
| **Dazap2** | 2 | 1 | 0 |
| **Rpia** | 0 | 1 | 0 |
| **Slc40a1** | 1 | 0 | 1 |
| **Txnip** | 0 | 0 | 2 |
| **Hp1bp3** | 1 | 1 | 0 |
| **2310007B03Rik** | 1 | 0 | 0 |
| **Entpd4** | 2 | 0 | 1 |
| **Thtpa** | 0 | 2 | 0 |
| **Lefty2** | 0 | 1 | 0 |
| **Tmem199** | 1 | 0 | 0 |
| **9030619K07Rik** | 4 | 0 | 0 |
| **Oasl2** | 0 | 1 | 0 |
| **Hn1** | 1 | 0 | 1 |
| **Tnfaip1** | 0 | 1 | 1 |
| **Tgfbr2** | 1 | 3 | 0 |
| **Epha2** | 0 | 1 | 0 |
| **Tbcel** | 2 | 1 | 0 |
| **Crct1** | 1 | 0 | 0 |
| **Tapt1** | 2 | 0 | 0 |
| **Mkrn1** | 1 | 0 | 1 |
| **Uchl1** | 0 | 1 | 1 |
| **Furin** | 1 | 0 | 0 |
| Hif1an | 1 | 2 | 1 |
| **Irf2** | 0 | 1 | 0 |
| **D030056L22Rik** | 1 | 0 | 1 |
| **Rnf213** | 0 | 0 | 1 |
| **Cdc2l6** | 2 | 2 | 0 |
| **Mmp14** | 1 | 0 | 0 |
| **Arhgef3** | 2 | 0 | 0 |
| **Pdk1** | 1 | 0 | 0 |
| **Zfp46** | 1 | 1 | 0 |
| **Ap1g2** | 0 | 2 | 0 |
| **Tes** | 1 | 0 | 0 |
| **Zfp367** | 2 | 1 | 1 |
| **Mcl1** | 0 | 0 | 1 |
| **Cdk2ap2** | 0 | 1 | 0 |
| **Irf1** | 1 | 0 | 0 |
| **Prdm4** | 0 | 1 | 0 |
| Cgn | 1 | 0 | 0 |
| **Syap1** | 0 | 0 | 2 |
| **St6galnac2** | 1 | 0 | 1 |
| **Slc35a4** | 0 | 1 | 0 |
| **Prkcdbp** | 0 | 1 | 0 |
| **Elavl2** | 0 | 1 | 1 |
| **BC037034** | 0 | 0 | 1 |
| **Sox11** | 2 | 0 | 0 |
| **9530068E07Rik** | 2 | 2 | 0 |
| **Specc1** | 3 | 1 | 0 |
| Klf6 | 1 | 1 | 0 |
| **Irak2** | 0 | 0 | 1 |
| **Irf2bp2** | 1 | 1 | 0 |
| **Bbx** | 1 | 0 | 0 |
| **Zkscan1** | 1 | 4 | 0 |
| **Clic1** | 2 | 0 | 0 |
| **Cenpq** | 0 | 1 | 0 |
| **Adfp** | 1 | 0 | 0 |
| **Pik3cb** | 2 | 0 | 0 |
| **Amotl1** | 3 | 0 | 0 |
| **Cxadr** | 1 | 0 | 1 |
| **Tmem183a** | 1 | 0 | 0 |
| Mark2 | 3 | 0 | 0 |
| Ndel1 | 3 | 0 | 0 |
| **Plagl2** | 1 | 1 | 1 |
| Blcap | 1 | 0 | 0 |
| **Pfn2** | 3 | 0 | 1 |
| **Ephb4** | 1 | 0 | 0 |
| **Zer1** | 0 | 2 | 0 |
| **Plekhm1** | 0 | 1 | 1 |
| **2410002F23Rik** | 0 | 1 | 0 |
| **LOC100044862** | 0 | 2 | 0 |
| **Arhgef5** | 1 | 0 | 0 |
| **Snx8** | 0 | 0 | 1 |
| Gabarap | 1 | 0 | 0 |
| Mll1 | 2 | 2 | 0 |
| **Bcl2l11** | 0 | 1 | 1 |
| **Abcc4** | 1 | 0 | 0 |
| **Zfp213** | 0 | 0 | 1 |
| **Nfib** | 0 | 1 | 0 |
| **Pml** | 1 | 0 | 0 |
| LOC100044475 | 0 | 1 | 0 |
| **Camk2n1** | 1 | 0 | 1 |
| **Mmp23** | 0 | 0 | 1 |
| **A230050P20Rik** | 0 | 1 | 0 |
| **Dctn4** | 3 | 1 | 0 |
| **1700019H03Rik** | 1 | 0 | 0 |
| Cep55 | 1 | 1 | 0 |
| Cyb561 | 1 | 0 | 0 |
| Plekhg5 | 1 | 0 | 0 |
| Ier3 | 0 | 1 | 0 |
| 2610301G19Rik | 0 | 1 | 0 |
| 4921505C17Rik | 1 | 1 | 0 |
| **Rragc** | 0 | 1 | 0 |
| **Fzd6** | 1 | 0 | 0 |
| 1500011K16Rik | 1 | 0 | 0 |
| **Dph2** | 0 | 1 | 0 |
| **2310002B06Rik** | 0 | 0 | 1 |
| **Hrbl** | 0 | 0 | 1 |
| Tagap | 1 | 0 | 0 |
| **Bcl7a** | 2 | 0 | 0 |
| Gns | 2 | 0 | 1 |
| Cc2d1a | 0 | 0 | 1 |
| **Ei24** | 0 | 1 | 0 |
| Centd1 | 1 | 0 | 0 |
| **Kif1b** | 0 | 1 | 0 |
| Tbc1d2 | 0 | 1 | 1 |
| **4933434E20Rik** | 1 | 1 | 0 |
| **Jmjd2b** | 1 | 0 | 0 |
| Aldh5a1 | 1 | 0 | 0 |
| 3110050N22Rik | 0 | 1 | 0 |
| Clptm1l | 0 | 1 | 0 |
| **Col18a1** | 0 | 1 | 0 |
| **Zbtb41** | 2 | 1 | 1 |
| Tnfrsf21 | 2 | 0 | 0 |
| Liph | 0 | 1 | 1 |
| **Muted** | 1 | 1 | 0 |
| Mmp9 | 1 | 0 | 0 |
| **Nt5e** | 1 | 0 | 0 |
| **Erbb2ip** | 1 | 0 | 1 |
| Dnajb9 | 2 | 0 | 0 |
| Tde2 | 0 | 1 | 0 |
| Mfn2 | 0 | 0 | 2 |
| **Zfp148** | 2 | 2 | 0 |
| **Hs6st1** | 1 | 1 | 0 |
| **Os9** | 0 | 1 | 0 |
| **BC025546** | 0 | 1 | 0 |
| Rbl1 | 0 | 0 | 1 |
| **Suv39h1** | 2 | 1 | 0 |
| Vim | 0 | 1 | 0 |
| **Crebl1** | 0 | 1 | 0 |
| **Anapc7** | 0 | 1 | 0 |
| Lace1 | 1 | 0 | 1 |
| Clip4 | 0 | 0 | 1 |
| Vps26 | 2 | 0 | 0 |
| Tmem184b | 1 | 1 | 0 |
| **Ddhd2** | 1 | 1 | 0 |
| **Tmem1** | 1 | 0 | 0 |
| Zfhx3 | 2 | 0 | 0 |
| BC013529 | 0 | 0 | 1 |
| E2f2 | 1 | 1 | 1 |
| Mgrn1 | 0 | 1 | 0 |
| Adam9 | 1 | 0 | 1 |
| **Ssx2ip** | 0 | 0 | 1 |
| Cacnb3 | 2 | 0 | 0 |
| Fzd10 | 0 | 1 | 0 |
| Golga1 | 1 | 1 | 0 |
| **Itm2b** | 1 | 0 | 0 |
| **March8** | 1 | 1 | 1 |
| 6030458C11Rik | 3 | 0 | 1 |
| Lrp2 | 0 | 1 | 0 |
| **C87436** | 1 | 1 | 0 |
| AA409316 | 0 | 1 | 1 |
| **Fbxo10** | 2 | 1 | 1 |
| **E330016A19Rik** | 1 | 2 | 0 |
| Lamp2 | 0 | 0 | 1 |
| **Rab15** | 1 | 0 | 0 |
| Arid2 | 1 | 0 | 0 |
| Zwint | 1 | 0 | 0 |
| 2700084L06Rik | 2 | 2 | 0 |
| Wwc2 | 2 | 0 | 0 |
| Phc2 | 1 | 0 | 0 |
| Grhl2 | 1 | 1 | 1 |
| LOC100048105 | 1 | 0 | 0 |
| Skil | 2 | 0 | 0 |
| LOC100044087 | 0 | 1 | 0 |
| Asb1 | 4 | 0 | 0 |
| **Arhgef18** | 1 | 0 | 1 |
| Zfp238 | 2 | 0 | 0 |
| **Coq10b** | 0 | 0 | 1 |
| Slc35b4 | 1 | 0 | 1 |
| Bicd2 | 2 | 1 | 0 |
| Rps6ka1 | 0 | 0 | 1 |
| **Itgb4** | 1 | 0 | 0 |
| BC043118 | 1 | 0 | 0 |
| Nme4 | 1 | 0 | 0 |
| **Snrk** | 1 | 0 | 0 |
| **Lrp11** | 0 | 0 | 1 |
| **Sbf1** | 0 | 1 | 0 |
| **2900037O03Rik** | 2 | 2 | 0 |
| Midn | 0 | 0 | 1 |
| Zfyve26 | 1 | 0 | 1 |
| Col4a2 | 0 | 0 | 1 |
| **Olig1** | 1 | 0 | 0 |
| Mtbp | 1 | 0 | 0 |
| AI661453 | 1 | 0 | 0 |
| **5830417I10Rik** | 1 | 0 | 0 |
| Gcnt2 | 0 | 1 | 0 |
| **Hs2st1** | 0 | 2 | 1 |
| Il17rd | 0 | 1 | 0 |
| **Cdca4** | 0 | 1 | 0 |
| 2310047D13Rik | 0 | 1 | 1 |
| **Leprotl1** | 0 | 1 | 0 |
| Rab7l1 | 0 | 1 | 0 |
| Stk11 | 0 | 0 | 1 |
| F2r | 1 | 0 | 0 |
| Stard10 | 1 | 0 | 0 |
| Phactr4 | 1 | 1 | 0 |
| LOC100046081 | 0 | 1 | 0 |
| **Hspb6** | 2 | 0 | 0 |
| Polk | 1 | 1 | 0 |
| Prdx3 | 0 | 1 | 0 |
| Ccdc100 | 1 | 0 | 0 |
| LOC100043906 | 0 | 0 | 1 |
| Stk17b | 2 | 0 | 0 |
| Gak | 1 | 0 | 0 |
| Tanc1 | 1 | 0 | 1 |
| 0710008K08Rik | 0 | 1 | 0 |
| AW112037 | 2 | 0 | 1 |
| Znfx1 | 3 | 1 | 1 |
| D10Ertd641e | 0 | 2 | 0 |
| Btg1 | 0 | 1 | 0 |
| Irf9 | 0 | 1 | 1 |
| Tctex1d2 | 1 | 0 | 0 |
| Necap1 | 0 | 1 | 0 |
| **Tmem129** | 0 | 1 | 0 |
| Dtx3l | 2 | 1 | 0 |
| 2010005O13Rik | 0 | 1 | 0 |
| Tmem111 | 0 | 1 | 0 |
| Avpi1 | 1 | 0 | 0 |
| Tesk1 | 1 | 0 | 0 |
| Mbnl1 | 2 | 0 | 0 |
| Fcho2 | 2 | 0 | 0 |
| 2310036O22Rik | 0 | 1 | 0 |
| Rnf26 | 0 | 1 | 0 |
| Slc30a3 | 1 | 0 | 0 |
| Usp42 | 0 | 1 | 0 |
| Kbtbd2 | 2 | 0 | 0 |
| Rsn | 1 | 0 | 0 |
| Zfp800 | 0 | 1 | 2 |
| Slain2 | 0 | 1 | 1 |
| AU022508 | 2 | 0 | 0 |
| Ccdc131 | 1 | 0 | 0 |
| Mkl2 | 2 | 0 | 0 |
| Mtus1 | 0 | 1 | 0 |
| Irgq | 1 | 0 | 0 |
| Tmem79 | 1 | 0 | 0 |
| LOC100044776 | 1 | 0 | 0 |
| Etv1 | 1 | 0 | 0 |
| Lpin1 | 0 | 1 | 0 |
| **Tagap1** | 1 | 0 | 0 |
| Trim56 | 0 | 2 | 0 |
| Cs | 0 | 1 | 0 |
| Osr1 | 1 | 0 | 0 |
| LOC100047707 | 0 | 1 | 0 |
| LOC100046844 | 0 | 2 | 0 |
| Pias3 | 1 | 0 | 0 |
| Prkd3 | 0 | 1 | 0 |
| Ptpn21 | 1 | 0 | 1 |
| Agps | 0 | 1 | 0 |
| Plekha3 | 1 | 0 | 0 |
| Map3k7ip1 | 0 | 1 | 0 |
| Pole3 | 1 | 0 | 1 |
| 1300001I01Rik | 0 | 1 | 0 |
| LOC676420 | 1 | 1 | 0 |
| Helb | 0 | 0 | 1 |
| Cyb5r1 | 0 | 1 | 0 |
| Rab32 | 1 | 0 | 0 |
| Xpr1 | 1 | 1 | 0 |
| Dpp8 | 0 | 1 | 0 |
| Spast | 1 | 0 | 0 |
| Pdcd5 | 1 | 0 | 0 |
| LOC100044170 | 1 | 0 | 0 |
| Aebp2 | 1 | 1 | 0 |
| Dync1li2 | 1 | 1 | 0 |
| Fjx1 | 1 | 0 | 0 |
| Pip4k2a | 0 | 0 | 1 |
| Unc13b | 1 | 0 | 0 |
| Wnt7b | 0 | 1 | 0 |
| Col4a1 | 1 | 0 | 0 |
| Ifitm3 | 1 | 0 | 0 |
| C330002I19Rik | 1 | 0 | 0 |
| Sipa1l3 | 0 | 0 | 1 |
| Cdc42bpb | 0 | 1 | 0 |
| 2700081O15Rik | 2 | 1 | 1 |
| Dgkq | 0 | 0 | 1 |
| Itga5 | 1 | 0 | 0 |
| LOC100046120 | 1 | 0 | 0 |
| Luzp1 | 2 | 1 | 0 |
| Rabgap1 | 1 | 0 | 1 |
| 1110039B18Rik | 0 | 1 | 0 |
| E2f1 | 2 | 0 | 0 |
| 2310051E17Rik | 1 | 0 | 0 |
| Serinc1 | 0 | 1 | 0 |
| Tinagl | 1 | 0 | 0 |
| Acaa1a | 0 | 0 | 1 |
| Lnx2 | 0 | 1 | 0 |
| Limk2 | 2 | 0 | 0 |
| Pkia | 1 | 1 | 0 |
| Whsc1 | 1 | 1 | 0 |
| Asb13 | 1 | 1 | 0 |
| Stx6 | 2 | 0 | 0 |
| D930001I22Rik | 1 | 1 | 0 |
| AU016693 | 1 | 0 | 0 |
| 1110021L09Rik | 1 | 0 | 0 |
| Zbtb7a | 4 | 0 | 0 |
| Skp2 | 1 | 1 | 0 |
| Neo1 | 0 | 2 | 0 |
| Dnajc16 | 2 | 2 | 0 |
| Sema4b | 1 | 0 | 0 |
| 2310004I03Rik | 0 | 1 | 0 |
| Arv1 | 0 | 0 | 1 |
| Sertad2 | 2 | 0 | 1 |
| 2310047A01Rik | 0 | 1 | 0 |
| 4732496O08Rik | 0 | 0 | 1 |
| Tmem68 | 0 | 1 | 0 |
| Nagk | 1 | 0 | 0 |
| Pdik1l | 4 | 1 | 0 |
| Arhgap27 | 0 | 1 | 0 |
| 4732462B05Rik | 1 | 0 | 0 |
| Fbxo45 | 2 | 0 | 0 |
| Tceb3 | 0 | 0 | 1 |
| Klhl21 | 0 | 1 | 1 |
| AL022832 | 3 | 0 | 0 |
| Fgf15 | 1 | 0 | 0 |
| Galnt10 | 0 | 1 | 0 |
| Kctd10 | 0 | 1 | 0 |
| Ap1s1 | 0 | 1 | 0 |
| 9330180L10Rik | 3 | 1 | 0 |
| Tmem55b | 1 | 0 | 0 |
| Nek9 | 2 | 0 | 1 |
| Kif23 | 1 | 0 | 0 |
| Wdr1 | 0 | 1 | 0 |
| AU040829 | 0 | 0 | 1 |
| 5730472N09Rik | 1 | 0 | 1 |
| Ap2b1 | 1 | 0 | 0 |
| Camk2n2 | 1 | 0 | 0 |
| Pdk4 | 0 | 0 | 1 |
| Adarb1 | 2 | 2 | 0 |
| Flvcr1 | 1 | 0 | 0 |
| Zmynd11 | 0 | 1 | 0 |
| Luc7l2 | 0 | 1 | 0 |
| Ncor1 | 1 | 0 | 0 |
| Limk1 | 1 | 0 | 0 |
| LOC100044636 | 1 | 0 | 0 |
| Csnk1g1 | 1 | 3 | 0 |
| Agpat3 | 1 | 0 | 0 |
| 1110011C06Rik | 0 | 1 | 0 |
| Rnf11 | 1 | 0 | 0 |
| A930008A22Rik | 0 | 1 | 0 |
| Znf512b | 3 | 0 | 1 |
| Ldoc1 | 1 | 0 | 1 |
| Gabrb3 | 1 | 0 | 0 |
| LOC100044557 | 1 | 0 | 0 |
| Zfp704 | 2 | 0 | 1 |
| Gtpbp2 | 1 | 1 | 0 |
| Mknk2 | 1 | 0 | 1 |
| Stk38l | 1 | 0 | 1 |
| Rrm2b | 0 | 1 | 0 |
| Ankrd17 | 0 | 0 | 1 |
| Senp7 | 0 | 2 | 0 |
| Scamp5 | 2 | 0 | 0 |
| Foxj3 | 3 | 0 | 0 |
| Grm6 | 2 | 0 | 0 |
| Eya3 | 1 | 1 | 0 |
| Fbxl14 | 1 | 0 | 0 |
| Mmp11 | 1 | 0 | 0 |
| Cirbp | 0 | 1 | 0 |
| Kctd3 | 1 | 0 | 0 |
| Rhod | 0 | 1 | 0 |
| Laptm4a | 1 | 0 | 0 |
| Arid4a | 2 | 1 | 0 |
| Ccng2 | 1 | 0 | 0 |
| Clock | 4 | 0 | 1 |
| Fbxo25 | 1 | 0 | 0 |
| Setx | 1 | 0 | 0 |
| Pvr | 1 | 0 | 0 |
| Hadhb | 0 | 1 | 0 |
| Zbtb5 | 0 | 2 | 0 |
| BC043098 | 0 | 1 | 0 |
| C230093N12Rik | 3 | 0 | 1 |
| Ftsj1 | 1 | 0 | 0 |
| Ing4 | 1 | 0 | 0 |
| Wasf2 | 1 | 0 | 0 |
| Zfp236 | 1 | 0 | 0 |
| Pigq | 2 | 0 | 0 |
| Ankmy2 | 0 | 0 | 1 |
| Ugcg | 1 | 1 | 0 |
| Add1 | 0 | 1 | 0 |
| Hapln3 | 2 | 0 | 0 |
| R3hdm2 | 0 | 1 | 0 |
| Nhsl1 | 1 | 0 | 0 |
| Fads2 | 0 | 1 | 0 |
| Tfip11 | 0 | 0 | 1 |
| Ifngr2 | 1 | 1 | 0 |
| Zfand2a | 1 | 3 | 1 |
| Adam17 | 0 | 1 | 0 |
| Rassf3 | 1 | 0 | 0 |
| Dock5 | 1 | 0 | 0 |
| LOC100047260 | 1 | 1 | 0 |
| Stambp | 0 | 1 | 0 |
| Fbxl4 | 0 | 0 | 1 |
| Tdrd7 | 1 | 0 | 0 |
| Csrp2 | 0 | 1 | 0 |
| Egfr | 1 | 0 | 0 |
| Tle4 | 0 | 0 | 1 |
| E430005I09Rik | 2 | 0 | 0 |
| Taf9b | 2 | 2 | 0 |
| Zmpste24 | 0 | 1 | 1 |
| Sec11a | 1 | 0 | 0 |
| Bahd1 | 1 | 1 | 0 |
| Tmcc1 | 1 | 0 | 0 |
| Alg2 | 0 | 1 | 0 |
| Dak | 0 | 1 | 0 |
| Map3k11 | 0 | 0 | 1 |
| Setd7 | 1 | 0 | 0 |
| Erbb2 | 0 | 1 | 0 |
| Dsn1 | 0 | 1 | 0 |
| Sept2 | 2 | 0 | 0 |
| Arhgap1 | 2 | 0 | 0 |
| Nit1 | 1 | 0 | 0 |
| Tmem50b | 1 | 0 | 0 |
| 5730596K20Rik | 1 | 0 | 0 |
| Smarcc2 | 0 | 1 | 0 |
| Fyco1 | 1 | 1 | 1 |
| Rhox6 | 0 | 1 | 0 |
| Zfp707 | 1 | 0 | 0 |
| Cbara1 | 0 | 0 | 1 |
| Epb4.9 | 2 | 0 | 0 |
| Slc39a6 | 0 | 1 | 0 |
| Irak3 | 1 | 0 | 0 |
| Ssr2 | 1 | 0 | 0 |
| Wrn | 1 | 0 | 0 |
| Lbxcor1 | 0 | 0 | 1 |
| Josd1 | 0 | 0 | 1 |
| App | 0 | 0 | 1 |
| Otub1 | 0 | 1 | 0 |
| 4930522L14Rik | 0 | 1 | 0 |
| Herc2 | 2 | 0 | 0 |
| Sp6 | 0 | 1 | 0 |
| Ppp2r2d | 1 | 0 | 0 |
| D330028D13Rik | 0 | 0 | 1 |
| Slc19a2 | 1 | 1 | 0 |
| Usp3 | 1 | 0 | 0 |
| A630054L15Rik | 1 | 0 | 0 |
| Cd109 | 0 | 1 | 0 |
| Bet1l | 0 | 0 | 1 |
| Ptpn14 | 1 | 0 | 0 |
| Tbc1d2b | 1 | 1 | 0 |
| AI837181 | 1 | 0 | 0 |
| Mcat | 0 | 1 | 0 |
| Tob2 | 1 | 0 | 1 |
| Rcan3 | 1 | 0 | 0 |
| 2900057K09Rik | 1 | 1 | 0 |
| Znrf1 | 2 | 0 | 0 |
| 4930402H24Rik | 0 | 2 | 0 |
| Hcfc1 | 2 | 1 | 0 |
| 5730410I19Rik | 1 | 0 | 0 |
| Kti12 | 1 | 0 | 0 |
| Map3k8 | 1 | 0 | 0 |
| Sft2d2 | 0 | 1 | 0 |
| Sept1 | 1 | 0 | 0 |
| Clk4 | 0 | 2 | 0 |
| D930048N14Rik | 2 | 0 | 1 |
| Tusc1 | 1 | 0 | 0 |
| Nfatc3 | 0 | 1 | 0 |
| LOC380860 | 2 | 0 | 0 |
| Ppp6c | 0 | 0 | 1 |
| Rasa1 | 1 | 0 | 0 |
| Ccdc137 | 0 | 1 | 1 |
| Casc4 | 0 | 0 | 1 |
| Dscr1l2 | 1 | 0 | 0 |
| Fbxo28 | 2 | 1 | 0 |
| Slc4a7 | 1 | 0 | 0 |
| Btaf1 | 0 | 1 | 0 |
| Pwwp2b | 1 | 0 | 0 |
| Ndn | 1 | 0 | 0 |
| Il6st | 1 | 0 | 1 |
| 1110048D14Rik | 0 | 1 | 1 |
| Cds1 | 2 | 0 | 0 |
| Camta1 | 0 | 0 | 1 |
| Zfp68 | 0 | 1 | 0 |
| Sirt4 | 0 | 0 | 1 |
| Tmem88 | 1 | 0 | 0 |
| Rab5c | 1 | 0 | 0 |
| Dmrta2 | 1 | 0 | 0 |
| Dock9 | 0 | 1 | 0 |
| 2210411K11Rik | 1 | 0 | 0 |
| Foxm1 | 2 | 0 | 0 |
| Sec63 | 2 | 0 | 0 |
| Mxd3 | 0 | 1 | 0 |
| Ctdsp2 | 1 | 0 | 0 |
| Tmem23 | 1 | 0 | 0 |
| 1700021K19Rik | 1 | 1 | 1 |
